# The pathogenesis of zoonotic viral infections: Lessons learned by studying reservoir hosts

**DOI:** 10.3389/fmicb.2023.1151524

**Published:** 2023-03-28

**Authors:** Lineke Begeman, Debby van Riel, Marion P. G. Koopmans, Thijs Kuiken

**Affiliations:** ^1^Viroscience, Erasmus University Medical Centre, Rotterdam, Netherlands; ^2^Pandemic and Disaster Preparedness Centre, Rotterdam, Netherlands

**Keywords:** zoonoses, comparative pathology, disease reservoirs, viruses, comparative histology

## Abstract

Zoonotic viral infections that cause severe disease or even death in some people may be asymptomatic or mild in reservoir hosts. Comparison of the pathogenesis of these two host categories may potentially explain the difference in disease. However, infections in reservoir hosts are often neglected. Therefore, we compared the pathogenesis of rabies virus, macacine alphaherpesvirus, West Nile virus, Puumala orthohantavirus, monkeypox virus, Lassa mammarenavirus, H5N1 highly pathogenic avian influenza, Marburg virus, Nipah virus, Middle East respiratory syndrome, and simian/human immunodeficiency viruses in both humans and reservoir hosts. We showed that most aspects of the pathogeneses were remarkably similar. The remaining differences lead to the identification of tipping points in the pathogeneses that are important for explaining the disease outcome in severe human cases. Further elucidating these tipping points by studying zoonotic viral infections in their reservoir hosts may teach us how to reduce the severity of zoonotic viral diseases in humans.

## 1. Introduction

Emerging infectious diseases cause severe disease in people and can disrupt societies, cost billions, and have the potential to become a pandemic (Taylor et al., 2001; Jones et al., 2008; Karesh et al., 2012; Gortazar et al., 2014; Carroll et al., 2018). This has been demonstrated by the COVID-19 outbreak. Reviews of past outbreaks have led to the assessment that the majority of emerging infectious diseases in humans come from animals, often from wildlife (Jones et al., 2008; Wang and Crameri, 2014), and that the majority are caused by viruses (Woolhouse et al., 2005). Mechanisms underlying (viral) emergence include human-induced changes in interspecies contacts through trade and habitat destruction (Gortazar et al., 2014; Plowright et al., 2017). Bats and rodents being frequent original hosts of zoonotic viruses could well be due to the species richness of these mammalian orders (Mollentze and Streicker, 2020).

A key question after a spillover of a virus from an animal reservoir is whether it is able to transmit between humans, thus leading to expanding outbreaks. A second key question is what the potential impact will be, which is related to the virus’s ability to cause disease in humans (Munster et al., 2020). The combined properties, transmissibility, and virulence define the eventual impact, and assessing them requires a combination of ecological, epidemiological, clinical, and pathological studies. Here, we focus on pathogenesis: understanding how the causative virus makes people ill. The study of pathogenesis is important for many aspects of disease control, including treatment, development of vaccines and antivirals, and prevention of virus transmission. The pathogenesis of viral infection can be studied in humans, reservoir hosts, and laboratory animals. However, the term “pathogenesis”, by itself, is a rather generic term that does not do justice to the differences between host species in the infection pathways and the mechanisms by which viruses can cause disease. When comparing the pathogenesis of a viral infection in two species groups, the generic term ‘pathogenesis’ becomes confusing. Therefore, we introduce new terminology here. Because the zoonotic viral infections studied here are “new” or spill-overs in the human host, we call their pathogenesis in humans “neopathogenesis”. We distinguish this from the original pathogenesis in the reservoir host to which the virus has adapted, which we term “orthopathogenesis”, derived from the word “ortho” meaning “correct”, and the pathogenesis observed in animals used to model the human disease in an experimental setup, which we term “parapathogenesis”, derived from the word ‘para’, meaning alongside.

In general, the pathogenesis of a viral infection can be split into different parts according to the pathway the virus follows in its host. From the perspective of a pathologist, using light microscopy, we can split them into (1) attachment to receptors on exposed host cells, (2) replication in host cells at the entry site, (3) release from host cells with subsequent dissemination within the host to other replication sites, (4) replication in host cells at sites that are important for virus amplification, and (5) excretion from the host to enable transmission to a new host. Together, these processes determine cellular, tissue, and immune responses, which in turn contribute to the clinical outcome of infection and the ability to spread viruses to the environment and new hosts. Because of ethical and practical reasons, we often cannot study the pathogenesis of an emerging viral disease directly in patients and instead use laboratory animal models. Researchers tend to focus on finding the best animal models, which are those in which the pathogenesis in the laboratory animal closely reflects that in the human host, i.e., in which parapathogenesis mirrors neopathogenesis.

Relative to neo- and parapathogenesis, orthopathogenesis is often ignored (with rare exceptions, e.g., Kuzmin et al., 2017). There are several potential reasons for this. Reservoir hosts are frequently in our blind spot: being shy or nocturnal, they generally live outside our vision; there is a relative lack of knowledge about their biology, at least among researchers that study human viruses, and frequently, the surveillance systems and the methods and materials to study them in the field and laboratory are not available. Furthermore, we tend to search for animal models that closely reflect the severe disease outcome in humans, while infection in reservoir hosts is often assumed to be asymptomatic. Nevertheless, we hypothesize that studying orthopathogenesis may be quite informative. It might be said that there is no better starting point for understanding virus pathogenesis than the reservoir host in which it has evolved. Examining pathogenesis in the reservoir host could eventually teach us at which stage the pathogenesis runs amok in people, leading to severe disease. This could provide us with the starting point for novel therapeutic targets to improve the outcome in severe human cases (Irving et al., 2021). This study aims to discover whether insights into the development of severe human disease can be derived from a comparison between the orthopathogenesis and neopathogenesis of some relatively well-studied zoonotic viral infections.

## 2. Materials and methods

**Figure 1:**
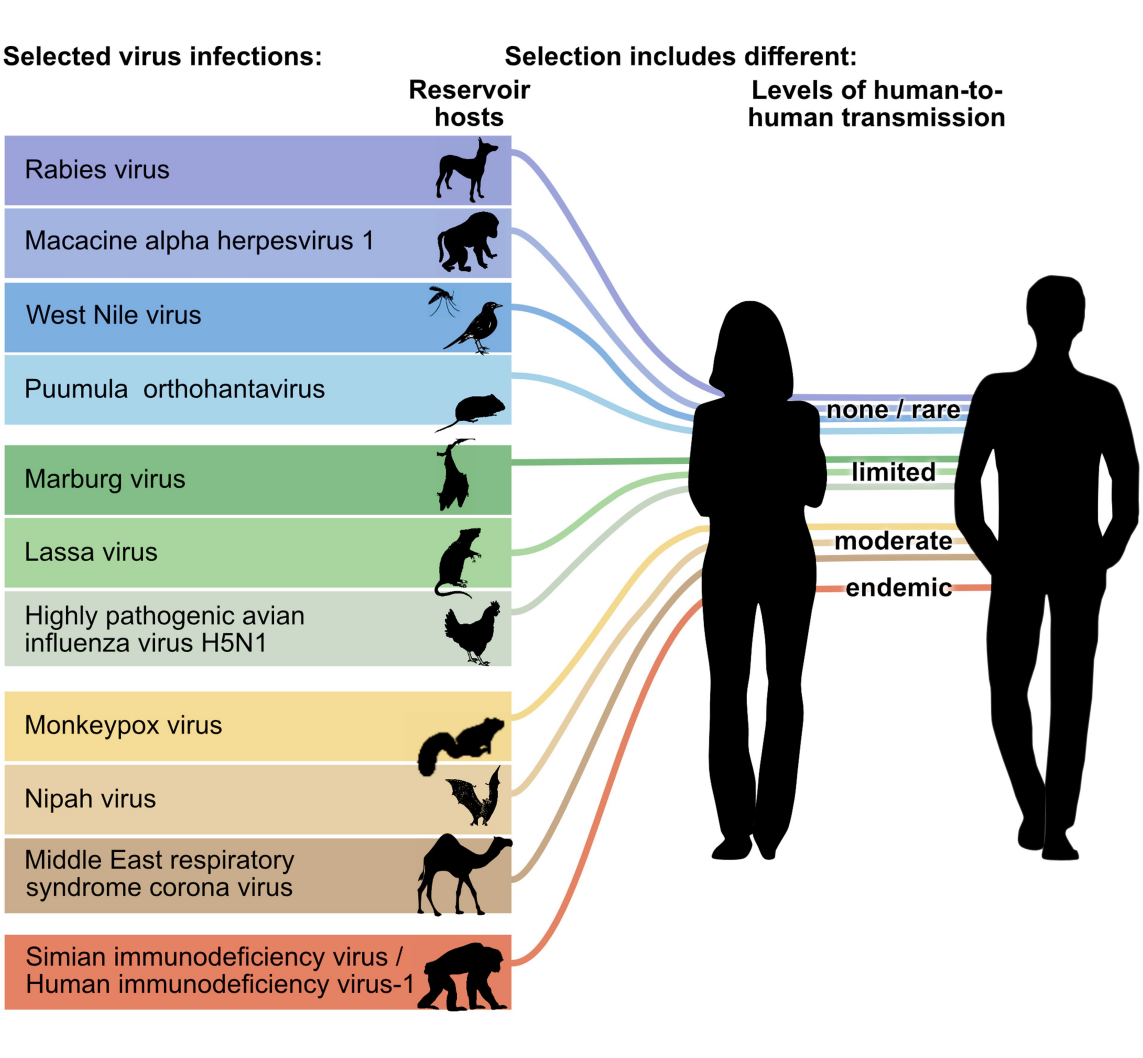
Zoonotic viruses selected for comparison between orthopathogenesis and neopathogenesis. Viruses represent different groups of reservoir hosts (orders of rodents, bats, carnivores, primates or ungulates, or classes of birds) and different stages of transmissions to humans (level of human-to-human transmission adapted from Wolfe et al., 2007). Please see the Table 1 for common and scientific names and mammalian order or avian class of reservoir host species schematically represented here. The mosquito is used to indicate that West Nile virus is an arbovirus, but the pathogenesis of the viral infection in mosquitos was not reviewed.

To learn more about potential insights that could be derived from a comparison of orthopathogenesis and neopathogenesis, we examined a series of zoonotic viral infections. We selected from a broad list of emerging infections. Our selection was limited to viral infections for which information was available regarding lesions and cell type or tissue tropism for both host groups, and, therefore, do not comprise a random selection. Reservoir host species have not been identified for some high-impact zoonotic diseases like those caused by SARS-CoV-1, SARS-CoV-2, and Ebola virus infections, and there are no data on the pathogeneses in suspected reservoir host species. Because of this lack of knowledge, these diseases could not be included. Because medical research generally focuses on zoonotic infections causing severe human disease, this list is biased toward viruses that are virulent for humans. We also selected viral infections to include reservoir host species of the most important orders of birds and mammals that harbor zoonotic viruses (Olival et al., 2017a,b) and to include different levels of transmissibility among humans (Plowright et al., 2017; Figure 1). For each of these zoonotic viral infections, we compared the orthopathogenesis with the neopathogenesis or, in the absence of knowledge of the neopathogenesis, the parapathogenesis.

**Figure 2:**
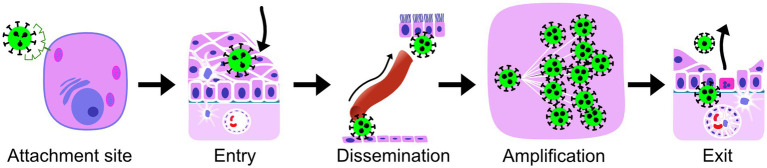
The virus-host interactions, compared for reservoir hosts and humans (Supplementary material), and the short terms for them used throughout the manuscript. *Attachment site*, the site on the outside of the host cell, where the cellular molecules (receptors) are located that the virus uses to attach to the host cells; *Entry*, the cell type or tissue that is used by the virus to enter a new host and is at the site of the start of the infection; *Dissemination*, the most important route of spread from the site of the start of the infection to the exit cell type, the route that is essential for further amplification and completion of the intra-host viral infection cycle, which can be blood, lymph (either within cells or free in the fluid), or interconnected neurons; *Amplification*, the main cell type in which amplification of virus takes place within the host; *Exit*, the cell type from which virus is excreted to infect a new host of the same species; The outcome of a viral infection for the health of the host is the overall result of virus-host interactions and the host immune responses.

For these selected zoonotic viral infections, we outlined some basic virus-host interactions at the tissue level, following the natural course of a viral infection (Figure 2). We focused on virus-host interactions that are relatively easily studied using a light microscope. Lesions can be visualized and characterized by staining tissues with hematoxylin and eosin. Viral proteins can be visualized within cells of those tissues using immunohistochemistry, while viral RNA can be visualized by *in situ* hybridization. These techniques allow for the co-localization of a virus with lesions and the identification of the cell tropism. Therefore, the five virus-host interactions we focused on were: (1) virus attachment site, (2) primary replication site or entry (cell types or tissue at the start of infection), (3) route of dissemination within the host, (4) virus amplification (cell types or tissues), and (5) cell types or tissues responsible for virus excretion. We also reviewed the outcome of infection, which can be seen as the end result of these virus-host interactions. Virus attachment site and entry were assessed separately because virus attachment is such a critical step, and they can be studied separately. For example, virus attachment sites can be studied on formalin-fixed paraffin-embedded tissue sections with virus histochemistry or receptor-specific immunohistochemistry of uninfected, healthy individuals, while entry can be studied by the examination of tissues from infected individuals.

The selected zoonotic viral infections belong to 11 different virus genera and 8 different virus orders: rabies virus (genus Lyssavirus, order Mononegavirales), macacine alphaherpesvirus 1 (genus Simplexvirus, order Herpesvirales), West Nile virus (genus Flavivirus, order Amarilloviralis), Puumula orthohantavirus (genus Orthohantavirus, order Bunyaviralis), Marburg virus (genus Marburgvirus, order Mononegaviralis), Lassa virus (genus Mammarenavirus, order Bunyaviralis), highly pathogenic avian influenza virus H5N1 (genus Alphainfluenzavirus, order Articulaviralis), monkeypox virus (genus Orthopoxvirus, order Chitovirales), Nipah virus (genus Henipavirus, order Mononegavirales), Middle East respiratory syndrome (MERS) coronavirus (genus Betacoronavirus, order Nidovirales), Simian/Human immunodeficiency virus-1 (genus Lentivirus, order Ortervirales) (Figure 1; Supplementary material). They are at five different levels of transmissibility amongst humans, ranging from not transmissible to endemic. This last category (endemic) includes a disease that was once a zoonosis, but during the time that it has been endemic in the human population, it has evolved into a virus that is not considered a zoonosis anymore. The majority of viruses, nine, are single-stranded RNA viruses, and the remaining two are double-stranded DNA viruses. Of the nine single-stranded RNA viruses, five are negative sense, three are positive sense, and one is ambisense. The reservoir hosts originate from five mammalian orders (Rodentia, Chiroptera [bats], Primates, Carnivora, and Artiodactyla) and two avian orders (Galliformes, Passeriformes) (Table 1).

**Table 1:** List of characteristics and selected reservoir hosts for the 11 selected viral infections included in the comparison.

Virus for which infection was compared				Reservoir host for which infection was compared to human host	
	Genetic structure	Genus	Order	Species (group)	Order
Rabies	RNA, single-stranded, negative sense	Lyssavirus	Mononegavirales	Carnivores	Carnivora
Macacine α herpes-1	DNA, double-stranded	Simplexvirus	Herpesvirales	Macaques	Primates
West Nile	RNA, single-stranded, positive sense	Flavivirus	Amarillovirales	Amplifying bird species*	Passeriformes
Puumula orthohanta	RNA, single-stranded, negative sense	Orthohantavirus	Bunyavirales	Bank voles (*Clethrionomys glareolus*)	Rodentia
Monkeypox	DNA, double-stranded	Orthopoxvirus	Chitovirales	African rope squirrels (*Funisciurus* spp.)	Rodentia
Lassa	RNA, single-stranded, ambisense	Mammarenavirus	Bunyavirales	Natal multimammate rats (*Mastomys natalensis*)	Rodentia
H5N1 HPAI	RNA, single-stranded, negative sense	α-Influenzavirus	Articulavirales	Chickens	Galliformes
Marburg	RNA, single-stranded, negative sense	Filovirus	Mononegavirales	Egyptian fruit bats (*Rousettus aegyptiacus*)	Rodentia
Nipah	RNA, single-stranded, negative sense	Henipavirus	Mononegavirales	Flying foxes (*Pteropus spp.*)	Chiroptera
MERS	RNA, single-stranded, positive sense	β-Coronavirus	Nidovirales	Dromedaries (*Camelus dromedarius*)	Artiodactyla
SIV /HIV-1	RNA, single-stranded, positive sense	Lentivirus	Ortervirales	Common chimpanzees (*Pan troglodytes*)	Primates

*The comparison includes bird species that in general do not have a fatal infection but do have a productive infection. Most of our comparison was based on an experiment with American robins (*Turdus migratorius*).

The severity of disease can differ within subpopulations of a host species, e.g., obese male adults may have more severe disease than children. Thus, we needed to make a choice about which subpopulation we wanted to study. We chose differently for the reservoir host and the human host as we were interested in what the orthopathogenesis could teach us about how viruses and hosts have adapted to each other. Therefore, if known, for reservoir hosts, we chose the pathogenesis of the infections in the subpopulation within the reservoir host that is most important for the maintenance of the virus in the species (often mild disease) (Swinton et al., 2009). With regard to the neopathogenesis, we were interested, for medical reasons, to learn about people with severe illness; therefore, we chose the pathogenesis of the infection in hospitalized patients (often adults with comorbidities and always with severe disease).

## 3. Results

Several trends in similarities and differences could be identified from the comparison between orthopathogenesis and neopathogenesis for each of these 11 examples of zoonotic viral infections. Details on assigning rates of similarity are available in the Supplementary material, as are the specific references that provide the basis for this assessment. Overall, the level of similarity between orthopathogenesis and neopathogenesis decreased from left to right in the table, or in other words, from the start to the end of the infection (Table 2).

**Table 2:** Overview of the results of the comparison of zoonotic viral infections with those of the original hosts, based on the literature review in the Supplementary material.

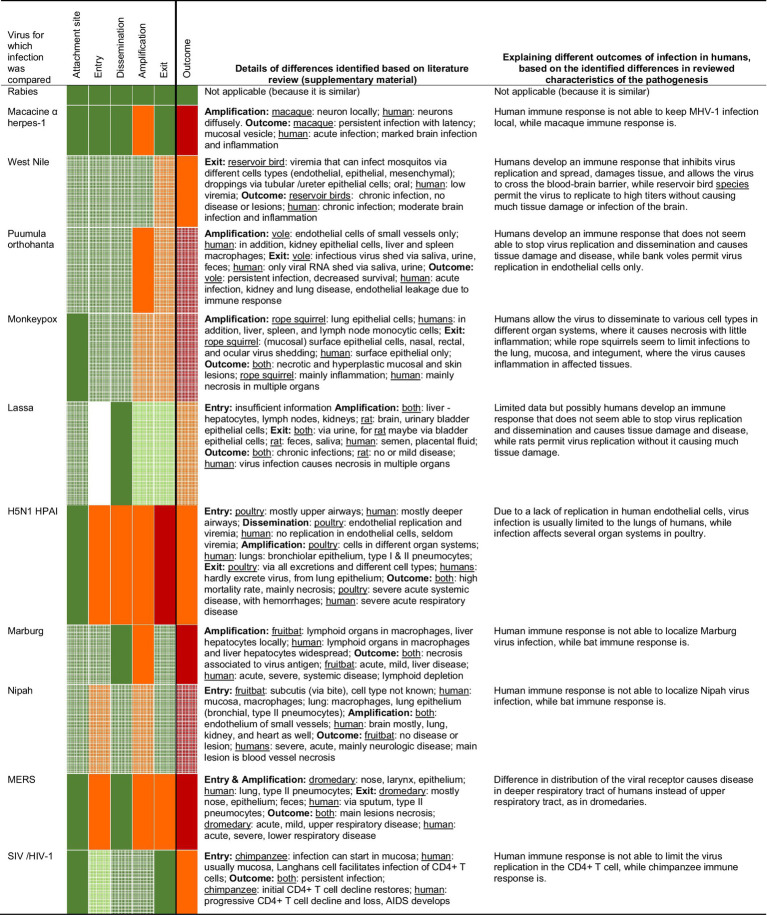

Dark green: similar; Lighter green: somewhat similar; Yellow: equivocal; Orange: somewhat different; Red: different; White grids indicate that available information is limited and, therefore, uncertainty exists regarding the human or reservoir host site of the comparison.

The first three virus-host interactions – virus attachment site, entry, and dissemination – were the most similar. First, the virus attachment site was generally the same for the reservoir host and humans. For example, the MERS virus attaches to dipeptidyl peptidase 4 (DPP4) and alpha-2,3-linked sialic acids in both dromedaries (*Camelus dromedarius*) and humans. Second, the entry was generally the same as well; this was assessed to be (likely) the same in 7 of the 10 examples for which we had information. Of note, even in two of the three examples in which entry was different (Middle East respiratory syndrome [MERS] and highly pathogenic avian influenza [HPAI] H5N1), the entry was still in the same organ system: the respiratory tract. However, the specific entry cell type was different: upper respiratory tract epithelial cells in reservoir hosts and lower respiratory tract epithelial cells in hospitalized humans. Third, dissemination of the virus was also well conserved, with 10 of the 11 examples being similar. When dissemination *via* blood or neurons was typical in the orthopathogenesis, this was also the case in the neopathogenesis. The exception was HPAI H5N1 in poultry, which can disseminate *via* blood abundantly because the virus can replicate well in galliform endothelial cells. In humans, however, endothelial cell infection and dissemination *via* blood occur less frequently, likely because of the lack of virus to replicate (well) in human endothelial cells *in vivo*. Thus, three of the five virus-host interactions were similar for the reservoir hosts and the human hosts for the majority (7 of 10 to 11 of 11) of our examples.

The two remaining interactions – amplification and exit – showed more differences between the reservoir host and human host: they were more frequently assessed to be (somewhat) different rather than (somewhat) similar. For amplification, 7 of the 11 infections were assessed as somewhat different. For four of those (macacine alphaherpesvirus 1, Puumula orthohantavirus, Marburgvirus, and Nipah virus infections), the differences arose not from the cell types themselves but rather from the number of infected cells, so it was a quantitative rather than a qualitative difference. The differences in the number of infected cells were inferred from reviewed literature in which quantities of cells were usually categorized, e.g., ‘few’ or ‘abundant’. In these four examples, humans showed infection in a larger proportion of cells than reservoir hosts. For the remaining three (monkeypox virus, HPAI H5N1, and MERS), the main cell type involved in the amplification phase in the reservoir host was also frequently important in the human host, but in the human host, the amplification also extended to other cell types. For exit, 5 of the 11 infections were assessed as (somewhat) different. For two of those (West Nile virus and Puumula orthohanta virus infections), the same cell types were likely involved in both reservoir hosts and humans, but this resulted in detectable excretion of infectious virus only in the reservoir hosts, suggesting, again, a quantitative rather than a qualitative difference. For the remaining three (monkeypox virus, HPAI H5N1, and MERS), the main cell type involved in virus exit was different, for instance, *via* infected nose epithelium in reservoirs but not humans, as this was not a (main) site of infection. In all three cases, this resulted in humans rarely or not excreting virus, and reservoir hosts excreting virus frequently *via* multiple routes.

Despite the many similarities in the selected characteristics of pathogenesis in reservoir hosts and human hosts, the outcomes of infection were different for 10 of the 11 infections. For 9 of these 10 infections, the outcome was more severe for the human host, as one would expect due to our selection bias (see Materials and Methods). The exception was HPAI H5N1, for which infection in chickens causes more severe disease and higher mortality than in humans. For 1 of the 11 infections, rabies virus infection, the disease outcome was similar for carnivores and humans.

Explanations for the differences in the outcome of infection appeared to have three patterns, based on quantitative and qualitative descriptions observed by microscopic examinations in the reviewed literature. First, differences could arise due to higher numbers of cells becoming infected during the amplification phase in the neopathogenesis, while the cell types were generally the same; here, the difference was quantitative. This was the case for infections with Marburg virus, macacine alphaherpesvirus 1, Nipah virus, HIV-1, and possibly also monkeypox virus and Lassa virus, in which the higher number of cells infected in humans compared to reservoir hosts was associated with more severe disease. Second, differences could arise from differences in cell type tropism during the start of the infection; here, the difference was qualitative. This was the case for the HPAI H5N1 virus, which infects endothelial cells in chickens, and, thus, enables the virus to spread systemically. Conversely, the lack of endothelial cell infection in humans enables the host to contain the viral infection mainly within the respiratory system. It was also the case for MERS coronavirus, which infects the upper airways and, thus, causes an upper respiratory tract infection in dromedaries. Conversely, MERS coronavirus in humans infects the deeper airways and, thus, causes a lung infection, which leads to more severe disease. Third, differences could arise due to an increased immune response associated with the same or even smaller numbers of cells infected in the neopathogenesis compared to the orthopathogenesis, e.g., in West Nile virus and Puumula hantavirus infections. In these infections, the higher number of infiltrated immune cells observed in humans compared to reservoir hosts was associated with more severe disease.

Though sufficient information was available for our comparison, significant knowledge gaps relating to basic parts of orthopathogenesis were revealed. When there were gaps, our comparison was based on assumptions and was, therefore, less confident (‘likely’). Most of the gaps (cells with white grit in Table 2) were due to a lack of knowledge about viral infection in the reservoir host rather than in the human host. In particular, there was a lack of knowledge of the cell types involved at different stages of the infection. This was because histological analyses were rarely performed in studies of naturally or experimentally infected reservoir hosts. Some knowledge gaps were quite consistent for our selected examples (Table 3): type of host cell receptor, distribution of host cell receptor, and type of viremia (cell-free or intracellular).

**Table 3:** Knowledge gaps (indicated by letter or *) in the orthopathogenesis.

Virus for which infection was compared	Attachment site	Entry	Dissemination	Amplification	Exit	Outcome	Details (*) of parts of the orthopathogenesis that are unknown
Macacine α herpes −1	D						-
West Nile	T, D	*	V		*		*Entry*: potentially intradermal dendritic cells, not confirmed; *Exit:* cellular origin of virus shed in oral cavity
Puumula orthohanta	T, D		V		*	*	*Exit:* cellular origin of virus in saliva, urine, feces; *Outcome:* virus-related lesions
Monkeypox	T, D	*	C, V	*	*	*	*Entry:* tissue and cell type; *Amplification:* cell type tropism not confirmed in natural infections; *Exit:* cellular origin of virus in nose, eye, rectum; *Outcome:* lesions and outcome of natural infection
Lassa	D		V	*	*	*	*Entry, Amplification, Exit:* tissues and cell types involved not confirmed in natural infections; *Outcome:* lesions and cell types affected at end point
Marburg	T, D	*			*		*Entry:* identification of tissue in which macrophages usually become infected; *Exit:* cellular origin of excreted virus
Nipah	D	*	V	*	*		*Entry:* cell type where infection starts; *Amplification:* endothelial tropism not confirmed; *Exit:* cellular origin of excreted virus
MERS					*		*Exit:* cell type involved in fecal virus excretion
SIV/ HIV-1		*		*			*Entry:* usual tissue type where infection starts; *Amplification:* particular CD4+ T cell type infected

D, distribution of the host cell receptor; T, type of host cell receptor; V, type of viremia, whether viremia is cell-free or intracellular; C, how virus reaches circulation.

## 4. Discussion

From this comparison between the orthopathogenesis and neopathogenesis of the 11 arbitrarily selected zoonotic viral infections, three main insights can be derived. First, most aspects of pathogenesis in humans are remarkably similar to those in reservoir species. This is an important finding because it can help us to predict aspects of infections of potentially zoonotic pathogens. Second, the identified differences between ortho- and neopathogenesis can pinpoint specific aspects of the pathogeneses that can partly explain the severe outcome of infection in people (Table 2). Third, there are many gaps in our knowledge of very basic parts of pathogenesis in reservoir hosts (Table 3), which, if resolved, might improve our ability to explain severe disease in people.

Regarding severe outcomes of infection in humans, two of three explanations seem directly related to a difference in immune response. First, severe outcomes of infection due to Marburg virus, macacine alphaherpesvirus 1, and Nipah virus, for example, could be explained by more cells becoming infected in humans than in reservoir species. This might suggest that, in contrast to the reservoir host, the human host is incapable of containing the infection to a few cells; there seems to be a hyporeaction of the human immune system, including the intracellular anti-viral response. It would be interesting to learn whether this is indeed the case, and what the underlying mechanisms are. Second, severe outcome of infection due to West Nile virus and Puumula orthohantavirus, for example, could be explained by severe immunopathological changes associated with the same or even a smaller number of cells becoming infected in humans than in reservoir species (Medzhitov et al., 2012). This might suggest that the human host responds excessively as there is a hyperreaction of the human immune system. In other words, the problems in humans arise from mismatches between the pathogen’s immune evasive responses and the human’s immune response (Table 4). Studying the immune response in the reservoir hosts more closely could clarify what the underlying mechanisms are that influence the extent of the immune responses in both host groups. A similar conclusion has been drawn by others who have reviewed literature focused on differences in the immune responses of reservoir hosts and people (Bean et al., 2013).

**Table 4:** Mismatches between the pathogen’s immune evasive responses and the human’s immune response might underly differences in outcomes of zoonotic viral diseases.

Effect upon infection as visualized by microscopy	Hypothesized immune response mismatch underlying the difference in outcome	Examples of viruses in which this seems to occur
More cells becoming infected in humans than in reservoir species	Hyporeaction: lack of human host cells to contain infection compared to reservoir host species	Marburg virus Macacine alphaherpesvirus Nipah virus
Severe immunopathological changes associated with a similar or smaller number of cells infected in humans than in reservoir species	Hyperreaction: human host cells respond excessively to infection compared to reservoir host species	West Nile virus Puumula orthohantavirus

Aside from the aforementioned reasons, there are other reasons for studying orthopathogenesis. A better understanding of orthopathogenesis can lead to the recognition of specific virus characteristics and their selective advantages. For example, we concluded from a previous comparative study of lyssavirus infections that the start of infection after a bat bite, due to tooth length and biting force, was most likely the skin, while for carnivores, it was the skeletal muscle. Thus, we predicted adaptations of bat lyssaviruses to replicate in skin and adaptations of carnivore lyssaviruses to replicate in skeletal muscle. These predictions provided an explanation for the observation of differences in cell type tropism and clinical disease of bat-acquired human rabies as opposed to carnivore-acquired human rabies (Begeman et al., 2018). Furthermore, orthopathogenesis may inform us about how the virus will behave in humans. A virus that has a tropism for neuronal cells in its bat host, like Lagos bat virus, seems likely to remain neurotropic in the human host. To take this one step further, because a newly emerged zoonotic virus is at an early stage of adaptation in the human host, knowledge of the pathogenesis in its reservoir host, where it is at an advanced stage of co-evolution, will inform us about the possible future route of evolution that the virus will take in humans. An example is MERS coronavirus, which causes an upper respiratory tract infection in dromedaries and transmits easily from dromedary to dromedary (Adney et al., 2014). If MERS coronavirus, now infecting deeper airways in humans and poorly transmissible, adapts to the human upper respiratory tract, we would expect it to become as transmissible in humans as it is in dromedaries. Therefore, we predict that upper respiratory tract infection will be part of the future pathogenesis of MERS in humans. This could result in epidemiology in the human population similar to that observed for SARS-CoV-2 (Munster et al., 2020).

With two of the four authors being pathologists, we focused our review on those virus-host interactions that are relatively easily studied using a light microscope. It would be interesting to see what overall insights can be derived from comparisons performed by researchers from other disciplines, e.g., immunologists, virologists, and cell biologists, who focus on other aspects of virus-host interactions.

The literature review performed for this perspective showed a lack of detailed descriptions of natural infections in reservoir hosts. We think these knowledge gaps could be relatively easily filled. For example, virological studies to identify reservoir hosts of a newly emerged zoonotic virus could be complemented by sampling formalin-fixed paraffin-embedded tissues to allow for pathological studies to determine the orthopathogenesis of this viral infection, without the need for sacrificing additional animals.

Here, we have introduced new terminology relating to pathogenesis in order to make it easier to communicate our comparison. However, it might be appropriate to make further differentiations in the terminology, depending on the context. For example, although this was not taken into account here, neopathogenesis might differ when a zoonotic infection has been acquired directly from the reservoir host, or indirectly from another human host. If the two are compared, more detailed terminology might be appropriate, e.g., heterologous versus homologous neopathogenesis. Taking this further, it is a matter of future research at which point a zoonotic pathogen adapts to such a degree to its human host that we should no longer speak of neopathogenesis.

In conclusion, our comparison of zoonotic viral infections between reservoir hosts and humans suggests several common principles: orthopathogenesis is remarkably similar to neopathogenesis; there are more similarities at the early stages of the infection cycle than at later stages; and observed differences can partly explain the severe outcome of infection in people. Therefore, directing more attention toward reservoir hosts promises to be a potent tool in increasing our understanding of zoonotic viral infections in humans and should become common practice.

## Data availability statement

The original contributions presented in the study are included in the article/Supplementary material, further inquiries can be directed to the corresponding author.

## Author contributions

LB and TK contributed to the conception and design of the study, organized the database, and wrote the first draft of the manuscript. All authors contributed to the manuscript revision and read and approved the submitted version.

## Funding

TK and LB were supported by the Dutch ZonMw programme on non-alimentary zoonoses under grant agreement no. 522003002 (Zoonoses in the Night). TK was supported by European Union Horizon 2020 program grant agreement DELTA-FLU no. 727922. DVR is supported by a fellowship from the Netherlands Organization for Scientific Research (VIDI contract 91718308) and an EUR fellowship. MK is supported by NWO Stevin Prize.

## Conflict of interest

The authors declare that the research was conducted in the absence of any commercial or financial relationships that could be construed as a potential conflict of interest.

## Publisher’s note

All claims expressed in this article are solely those of the authors and do not necessarily represent those of their affiliated organizations, or those of the publisher, the editors and the reviewers. Any product that may be evaluated in this article, or claim that may be made by its manufacturer, is not guaranteed or endorsed by the publisher.

## Glossary

Introducing new terminology to aid in the discussion of differences between three different host groups in the pathogenesis of certain viral infections. For this paper we consider only humans as the novel host and only non-human vertebrate animals as reservoir hosts.: Neopathogenesis: pathogenesis of viral infection in novel host (host in which no or limited viral adaptation has occurred). Orthopathogenesis: pathogenesis of viral infection in natural reservoir hosts[.] Parapathogenesis: pathogenesis of viral infection in laboratory animal model used to model the disease of interest in an experimental set-up. For this paper we used the parapathogenesis when knowledge of neopathogenesis was not available.
