# Supplementary material for: The pathogenesis of zoonotic viral infections: Lessons learned by studying reservoir hosts
Source: Front Microbiol. 2023 Mar 28;14:1151524. doi: 10.3389/fmicb.2023.1151524 (PMC10086422; doi:10.3389/fmicb.2023.1151524)
Supplement: Supplementary file 1 [file Data_Sheet_1.docx]

**Supplementary Material**

**1. Rabies virus (negative sense, single-stranded RNA virus, genus Lyssavirus, order Mononegavirales) infection in carnivores (order Carnivora) versus rabies virus infection in humans**

**Attachment site:** Rabies virus has been shown to attach to nicotinic Acetylcholine receptor to enter myocytes, and to neuronal cell adhesion molecule or p75 neurotrophin receptor to enter neurons. It is assumed that these receptors (Lafon, 2005) are well conserved across the different host species.

**Entry:** In carnivores, rabies virus is mainly transmitted via bites. The infection starts in the neurons innervating the area of the bite. In addition, shortly after infection of the neurons, a small number of fibroblasts and skeletal myocytes can also become infected. The relevance of infection of fibroblasts and skeletal myocytes for the pathogenesis of rabies is not clear (Smart and Charlton, 1992, Charlton and Casey, 1981).

Humans acquire rabies virus infection mainly via a bite of an infected carnivore. Infection is assumed to start in the neurons innervating the area of the bite. This is based on studies in laboratory animals (rats, mice, and hamsters) that aimed to look at infection shortly after inoculation (Jackson and Fu, 2013). It has not been investigated whether human fibroblasts and skeletal myocytes can also become infected at an early stage during infection.

**Dissemination:** Both in carnivores and humans, rabies virus disseminates from the neurons at the location of the bite, via synapses, to other neurons and so spreads to the central nervous system.

**Amplification:** After dissemination, in both carnivores and humans, rabies virus continues to spread among neurons in the central nervous system because the immune system does not stop replication (Schnell et al., 2010). Thus, rabies virus mainly replicates in neurons (Rossiter, 2013).

**Exit:** Both carnivores and humans excrete virus in the oral cavity, via infection of minor or major salivary glands. Both serous and mucous gland epithelial cells are infected in the salivary glands. In addition, the epithelial cells of the tongue are likely involved in virus excretion, both in humans and carnivores (Li et al., 1995, Shiwa et al., 2018).

**Outcome:** In carnivores and humans, rabies causes a severe neurological disease with a case fatality rate of nearly 100%. At the time of death there is abundant presence of virus antigen in neurons throughout the whole nervous system. In both carnivores and humans there is neuronal loss and a relatively mild infiltrate composed of mainly lymphocytes and fewer plasma cells in the nervous system (Rossiter, 2013).

**2. Macacine alphaherpesvirus 1 (Cercopithecine herpesvirus 1, Herpesvirus simiae, Monkey B virus; double-stranded DNA virus, genus Simplexvirus, order Herpesvirales) infection in macaques (*Macaca spp*. order Primates) versus Macacine alphaherpesvirus 1 infection in humans**

**Attachment site:** Macacine alphaherpesvirus 1 attaches to nectin-1 and -2 receptors on cells of both macaques and humans. Nectins are proteins that are ubiquitously expressed. They have a function in cellular adhesion of epithelial and endothelial cells, and are part of the chemical synapse of neuronal cells (Eberle and Jones-Engel, 2018). There are species differences in nectin proteins. It is not known whether these species differences in the nectin proteins influence the pathogenesis of the infection in macaque and human hosts (Patrusheva et al., 2016).

**Entry:** In macaques, macacine alphaherpesvirus 1 is mainly transmitted via direct contact. The infection starts in the mucosal epithelial cells of the site exposed (mainly oral mucosa if first acquired at a young age, and genital mucosa if acquired as an adult) (Eberle and Jones-Engel, 2018, Bailey and Miller, 2012).

Humans acquire macacine alphaherpesvirus 1 infection mainly via a bite of an infected macaque. In these cases, the infection likely starts in the epithelial cells in the area of the bite. This is based on the occurrence of vesicular herpetic lesions at the site of exposure (Eberle and Jones-Engel, 2018), and the permissiveness of human epithelial cells for the virus (Perelygina et al., 2015). Infection can also start in mucous membrane epithelial cells, when direct contact with infectious macaque material occurs, e.g. during a laboratory exposure (Eberle and Jones-Engel).

**Dissemination:** In macaques macacine alphaherpesvirus 1 disseminates from the epithelial cell to sensory neuron endings innervating the epithelium. Virus is transported in the axons of the sensory neurons, to the cytoplasm and nucleus of those neurons. These are located in the dorsal root ganglia for the trunk and neck, and the trigeminal ganglion for the face (Eberle and Jones-Engel, 2018). In humans the infection is assumed to disseminate similarly to macaques, to sensory neuron ganglia (Eberle and Jones-Engel, 2018). This is based on the findings that at the end stage of the human infection, virus had widely disseminated to different neurons.

**Amplification:** In macaques, the virus replication is usually halted in the sensory neuron or neurons initially; the infection is then in a latent phase. When reactivation of the infection occurs, replication of virus leads to infection of mucosal epithelial cells of the area that is innervated by the infected neuron, rather than leading to infection of other neurons. In a minority of very young or immunosuppressed macaques, macacine alphaherpesvirus 1 infection does disseminate widely, causing areas of necrosis in the mucosal surface of the upper gastrointestinal tract, liver, pancreas, adrenal gland, spleen and lymph nodes. Herpes virus inclusions, indicating viral presence, have been detected in epithelial cells and syncytia of these tissues, in addition, viral replication in hepatocytes has been confirmed by electron microscopy. Strikingly, lesions were not consistently detected in the brainstem, cerebellum and cerebrum of macaques with disseminated infection. Spinal cord, when investigated, did have lesions (Carlson et al., 1997). These findings indicate that macacine alphaherpesvirus 1 can infect and replicate a wide variety of cell types in macaques. That this only occurs in a minority of immunosuppressed individuals suggests the immune response of the host usually prevents viral dissemination. In humans, virus is assumed to disseminate via neuronal connections and causes a widespread infection of neurons throughout the central nervous system. This is based on the clinical neurologic disease the infection causes in humans, and the finding of neuronal necrosis and presence of virus in the nervous system in the end stage disease of humans (Nanda et al., 1990). It has not been investigated whether macacine alphaherpesvirus 1 infects the epithelium of gastrointestinal tract, liver, pancreas and lymphoid organs in humans like it does in macaques with disseminated infections. In a minority of infected humans, macacine alphaherpesvirus 1 can, like in macaques, go into a latent phase, and reactivate weeks to years later (Eberle and Jones-Engel, 2018).

**Exit:** Infected macaques periodically (during viral reactivation) shed virus from infected mucosal epithelial cells, usually, but not always, accompanied by the formation of blisters (Bailey and Miller, 2012). Periodic shedding of macacine alphaherpesvirus 1 from infected areas has been described in humans (Eberle and Jones-Engel, 2018), presumably from infected mucosal epithelial cells.

**Outcome:** In macaques the virus infection causes a mucosal vesicle, or no lesion at all. In rare cases that immunosuppressed macaques have a disseminated infection, necrosis is present in many organs, including mucosal surfaces of the mouth, gastrointestinal tissues, liver, pancreas, adrenal glands, spleen and lymph nodes. Meningomyelitis is inconsistently present in spinal cord, and virus can be isolated from serum in some cases (Carlson et al., 1997). In humans the virus infection causes a marked meningoencephalomyelitis, characterized by haemorrhages, neuronal necrosis and marked infiltration with lymphocytes, with relative sparing of the cerebrum (Nanda et al., 1990). Dissemination to other organs than the brain occurs (Nanda et al., 1990), but description of the associated lesions and viral tropism could not be found in the literature. The disease is fatal in the majority of cases (CFR >50%). In mice, used to model the human disease brain, lesions were characterized by neuronal necrosis with axonal and neuropil loss mainly, and less mononuclear infiltration. Within the brain, lesions (as well as the infection) were confined to the brainstem. In the spinal cord lesions were mainly present in sensory tracts. In dorsal root (sensory) ganglia, there was mononuclear infiltration (Ritchey et al., 2005).

**3. West Nile virus (positive sense, single-stranded RNA virus, genus Flavivirus, order Amarillovirales) infection in amplifying bird species (here included are those bird species that in general do not have a fatal infection, but do have a productive infection), versus West Nile virus infection in humans**

**Attachment site:** It is not well recognized which receptor(s) on host cells are used by West Nile virus or other Flaviviridae for attachment and entry. In both birds and humans, the virus probably uses dendritic cell-specific ICAM-3 grabbing non-integrin (DC-SIGN) related receptors for entry into intradermal dendritic cells. There are species differences in these receptors (Gupta and Gupta, 2012). It is not known whether these species differences in the DC-SIGN related receptors influence the pathogenesis of the infection in bird and human hosts.

**Entry:** West Nile virus infection is transmitted to birds and humans mainly by (*Culex* sp.) mosquito bites. In birds and humans, the infection is assumed to start in the intradermal dendritic cells at the bite site (Gamino and Hofle, 2013).

**Dissemination:** In birds and humans, virus is thought to carried by the infected dermal dendritic cell into local lymph vessels, and from there into the blood circulation. It is not clear how virus disseminates from the circulating infected dendritic cell to other tissues and cells. Options are that dendritic cells first cause a cell free viraemia, which causes infection in cell types important for further amplification of virus, or that dendritic cells first emigrate from blood vessels into tissues where amplification cell types are infected (Gamino and Hofle, 2013, Perez-Ramirez et al., 2014).

**Amplification:** In birds, virus can replicate in a wide variety of cell types and organs, including dendritic cells and macrophages in the spleen, and neurons in the brain (VanDalen et al., 2013). It is not clear which cell type is most important for the high and sometimes prolonged viraemia. In humans, virus amplification likely occurs mainly within lymph nodes, spleen, and brain. The cell type tropism is not described for humans, but likely includes macrophages and neurons.

**Exit:** Birds can develop a chronic West Nile virus infection, during which the virus likely replicates in many different cell types (including epithelial, endothelial, and mesenchymal cells) in different organs (Gamino and Hofle, 2013, Perez-Ramirez et al., 2014). The infection induces a viraemia that is high enough, at least for a short period, to infect feeding mosquitoes, but the cell type mainly responsible for the viraemia is not known. Aside from this vector route of excretion, viral replication in kidney tubular epithelial cells, and ureter epithelial cells likely causes virus excretion via droppings. In addition, birds can shed infectious virus in the oral cavity, but the cellular origin for this virus is not clear. Oral shedding can occur from 2 to 14 days post inoculation, and in absence of a viraemia (VanDalen et al., 2013). In humans viraemia occurs, though it is not clear which cell types are mainly involved in virus production. The viraemia is too low to infect feeding mosquitoes. Viral RNA has been detected in convalescent humans, sometimes years after having West Nile virus disease, suggesting humans can become persistently infected, while they do not excrete infectious virus, nor have a viraemia that could infect feeding mosquitoes (Gamino and Hofle, 2013, Perez-Ramirez et al., 2014). The tissue and cell type in which virus can reside during a persistent infection are not known.

**Outcome:** Many different bird species can become lethally infected with West Nile virus. We chose to make this comparison with bird species that act as amplification hosts, with a non-fatal yet productive West Nile virus infection, meaning high viral loads in the blood (>10^6^ PFU/ml). These bird species usually do not show clinical signs. Virus replication likely occurs in many different cell types, in different organs initially, without inducing inflammation. Later during the infection, virus is less widespread, e.g. in American robins (*Turdus migratorius*), virus was only detected in goblet cells lining the villi and crypts in the intestine, and in epithelium of the ureter. A minority of this bird species dies of an infection (2 of 25 birds experimentally infected). In the birds that died, neurons in ganglia and neurons in the brain were infected (VanDalen et al., 2013). It was not clear whether the infection in the brain was associated with inflammation. In the majority of people, infection is asymptomatic. Of infected people, 20-30% develop flu-like illness. Only 0.7% develops neurologic illness, of which the case fatality rate is 10% (Samuel and Diamond, 2006). In these humans, virus has crossed the blood-brain barrier and infected neurons, which causes a (meningo)encephalomyelitis. The lesions are focused in the basal ganglia, thalamus, brainstem (medulla and pons) and anterior horn of the spinal cord, and are composed of glial nodules composed of lymphocytes and histiocytes. Infiltrates are mainly composed of CD8+ T cells, less CD4+ T cells, and CD20+ B cells surrounding blood vessels (Sampson et al., 2000). There are several hypotheses how virus passes the blood-brain barrier: peripheral production of tumor necrosis factor alpha leading to increased blood-brain barrier permeability; via infection of and migration across cerebral endothelial cells; via migration of West Nile virus infected leukocytes; via infection of choroid epithelial cells shedding virus into the cerebrospinal fluid; or via transport of West Nile virus from the peripheral nerve fibers to neuronal cell bodies in the central nervous system (Sejvar, 2016).

Our information on the pathogenesis of West Nile virus infection in avian amplification host species originated mostly from a study performed on an American amplification host, the American robin. The pathogen is relatively new on the American continent. It would be interesting to learn more about the pathogenesis in original hosts from the African continent, that have co-evolved with the virus much longer (Perez-Ramirez et al., 2014).

**4. Puumula orthohantavirus (negative sense, single-stranded RNA virus, genus Orthohantavirus, order Bunyavirales) infection in bank voles (*Clethrionomys glareolus*; order Rodentia), versus Puumula orthohantavirus infection in humans**

**Attachment site:** Puumula orthohantavirus likely uses integrin receptors and co-receptors to enter bank vole cells. Integrins are present on almost all vertebrate cells. The specific types of receptors are not known for both humans and bank voles (Muller et al., 2019, Hägele et al., 2021), although differences in receptor usage are believed to be one of the crucial determinants of pathogenicity (Gavrilovskaya et al., 2002). More detailed in vitro studies, with human or monkey cells or proteins, with different orthohantaviruses have described other proteins as (co-) receptors, protocadherin-1 (Jangra et al., 2018), decay-accelerating factor/CD55 (Krautkramer and Zeier, 2008), and the receptor for globular head domain of complement C1q/p32/p33 (Choi et al., 2008). A comparison with entry into cells from original hosts were not made.

**Entry:** Puumula orthohantavirus infection is transmitted to bank voles and humans by inhalation of virus-containing aerosols. The infection likely starts, for bank voles as well as for humans, in lung epithelial cells (not specified) and lung macrophages. In lungs, alveolar cells and macrophage-like cells have been shown to contain antigen in naturally infected bank voles (Brummer-Korvenkontio et al., 1980). For humans there is not data on the cell type involved in the start of infection (Noack et al., 2020). Also, in the macaque model used to model the human infection, the start of infection, or the aerosol route of transmission were not investigated. In the macaque model, after 28 days post intravenous inoculation, viral RNA was not detected in the lungs.

**Dissemination:** In bank voles and humans, potentially via infected lung macrophages, virus enters the blood, through which it disseminates. In bank voles viraemia (based on the detection of RNA in serum) occurs for months, if not life-long, and only shows a slow decline over time (Voutilainen et al., 2016). In humans viraemia (based on the detection of RNA in serum) occurs for a short time (days) usually (Evander et al., 2007), but can occur for three to four weeks in some cases (Mustonen et al., 2013).

**Amplification:** In general, for orthohantaviruses in the rodent host, virus mainly replicates in endothelial cells of smaller blood vessels, in lungs and kidneys, and in macrophages (Noack et al., 2020), and these cells can be expected to be the source for the viraemia. It is not clear whether endothelial cells become infected from a cell-free viraemia, or through direct contact with an infected macrophage. In humans that become ill, or in cynomolgus macaques used to model human disease, Puumula orthohantavirus replicates mainly in distal, and potentially also proximal, tubular epithelial cells in the kidneys, and can be detected in capillary endothelial cells in the kidneys, liver and spleen. Virus RNA has also been detected in Kupffer cells in the liver and in dendritic cells in the spleen (Sironen et al., 2008), although it is not clear if the virus can replicate in these cells.

**Exit:** Bank voles infected with Puumula orthohantavirus shed virus in saliva, urine and faeces for months, if not life-long (Voutilainen et al., 2016, Hardestam et al., 2008), potentially from infected endothelial cells, which induce viraemia, and subsequent leakage of virus from the systemic circulation into excreta. However, the sources of virus for different excretion routes have not been specifically investigated. Humans infected with Puumula orthohantavirus were tested for virus excretion in saliva after hospitalization, and RNA was detected up to 9 days after disease onset in 10 of 14 patients. The RNA positive saliva could not infect bank voles or Vero 6 cells and hence the presence of infectious virus in saliva was not shown (Pettersson et al., 2008, Godoy et al., 2009). The origin of this viral RNA in the saliva was not clear. There was no clear correlation between plasma RNA level and salivary RNA level in these patients, while there was a trend towards severity of respiratory illness being associated with an increased chance of salivary RNA detection. For a related orthohantavirus, Andes virus, viral antigen has been detected in salivary gland epithelial cells of infected people suggesting salivary virus could be produced in the salivary glands . In macaques infected with Puumula orthohantavirus virus antigen was detected in the lumen of tubuli in the kidney, and suggested to be a sign of virus excretion. However, urine was not tested for the presence of the virus (Sironen et al., 2008).

**Outcome:** In bank voles, the persistent Puumula orthohantavirus infection causes decreased winter survival and reduced body weights (Hardestam et al., 2008), for which the cause is not clear. Though the occurrence of lesions in Puumula-infected bank voles has not been investigated, for other orthohantavirus-infected rodents there is no, or limited inflammation associated with infection (Maas et al., 2019). In humans Puumula orthohantavirus infection may lead to nephropathia epidemica (case fatality rate of 1%), which is characterized by gastrointestinal discomfort, oliguria, proteinuria and later, polyuria and even hematuria. Virus antigen has been detected in human kidney biopsies in endothelial cells, tubular epithelial cells and podocytes of the glomeruli (Groen et al., 1996, Mustonen et al., 2017), and in human intestinal biopsies within endothelial cells within the lamina propria (Latus et al., 2014). Infected endothelial cells do not show any abnormalities, while tubular epithelial cells show necrosis. Kidney interstitium shows oedema, and is infiltrated with lymphocytes, plasma cells, monocytes and neutrophils, and haemorrhages can be present. The pathogenesis of the disease nephropathia epidemica is thought to be due to infection of endothelial cells in mainly kidneys causing increased permeability due to loss of function of infected endothelial cells, with preservation of the cells. The change in permeability of the endothelial barrier is thought to be due to the human innate immune response, with an increased immune response being correlated with an increased disease severity. Proteinuria is due to loss of function at the glomerular and tubular levels. Acute thrombocytopenia is also an important characteristic of the (early) disease, but it is not clear how it develops (Ermonval et al., 2016, Mustonen et al., 2017). Aside from the kidney-injury related disease, a minority of infected people in addition show respiratory disease, with coughing and dyspnea (Pettersson et al., 2008), for which the pathogenesis is not clear. In general, for orthohantaviruses causing human respiratory disease, the mechanism is suggested to be similar to the kidney-related disease, namely due to endothelial cell barrier dysfunction, secondary to the host-immune response (Sironen et al., 2008, Ermonval et al., 2016). In macaques infected with Puumula orthohantavirus, interstitial pneumonia, which includes lymphocytic infiltrates, were detected at 28 days post inoculation and were suggested to be due to the virus infection, while virus could not be detected at this stage anymore (Sironen et al., 2008).

**5. Marburg virus (negative-sense, single stranded RNA virus, genus Filovirus, order Mononegavirales) infection in Egyptian fruit bats (*Rousettus aegyptiacus*; order Chiroptera), versus Marburg virus infection in humans**

**Attachment site:** Marburg virus attaches to glycosaminoglycans and other surface cellular proteins (Salvador et al., 2013). Glycosaminoglycans are ubiquitously expressed by all mammalian cells, and have many functions including in cell adhesion and regulation of proliferation. The type and pattern on the cell surface of glycans are likely to be important for viral attachment. The type and pattern of glycosaminoglycans, and other host receptors that are relevant for Marburg virus infection, and the species differences between those in Egyptian fruit bats and humans, are not known.

**Entry:** In Egyptian fruit bats, Marburg virus is mainly transmitted via direct contact, potentially via oral intake of virus (Schuh et al., 2017). The infection likely starts in macrophages (Jones et al., 2019). Humans acquire Marburg virus infection via contact with infected bats or their excreta, or via contact with infected humans or their excreta. Similar to the bats, the first cells to become infected are likely macrophages. For both Egyptian fruit bats and humans it is not clear how and in which tissue the virus gets into macrophages.

**Dissemination:** In both Egyptian fruit bats and humans Marburg virus disseminates through the blood circulation (Jones et al., 2019, Schuh et al., 2017). In blood, virus is likely present within infected macrophages, as well as cell free (Shifflett and Marzi, 2019)

**Amplification:** In Egyptian fruit bats and humans, virus replication occurs mainly in macrophages in lymphoid organs, in hepatocytes and in fibroblasts (Jones et al., 2019, Schuh et al., 2017, Shifflett and Marzi, 2019).

**Exit:** In Egyptian fruit bats infected with Marburg virus, infectious virus has been recovered from saliva, urine and faeces. The cell type source for the virus in these excreta is not known (Jones et al., 2019, Schuh et al., 2017). In humans infected with Marburg virus, infectious virus has been recovered from all body fluids. The source of virus was likely leakage from the circulation, which contains a large amount of (cell-free) virus (Shifflett and Marzi, 2019).

**Outcome:** In Egyptian fruit bats, Marburg virus infection causes an acute, immunizing, very mild disease. The main lesion in bats occurs in the liver, and is associated with an increased liver enzyme detection in the blood. Lesions in the liver are characterized by randomly-scattered aggregates of macrophages and lymphocytes, with occasional neutrophils and variably necrotic, apoptotic, or degenerating hepatocytes and karyorrhectic cell debris associated to viral presence. Egyptian fruit bats seem to clear the infection, and become resistant to re-infection (Jones et al., 2019, Schuh et al., 2017). In humans, Marburg virus infection causes an acute severe systemic disease, with a case fatality rate of about 40%. At the start of infection there is severe gastrointestinal illness, for which the pathogenesis is not well explained. Later a maculopapular rash can develop, and spontaneous bleedings can occur. In later stages neurologic symptoms can occur. At the time of death necrotic foci and haemorrhages are present in all tissues examined, and associated with cells containing viral inclusions. Necrosis is marked in liver, lymph nodes, testis and ovaries (Shifflett and Marzi, 2019). Lymph nodes show lymphoid depletion, associated with apoptosis. The apoptosis is not a direct effect of the virus, as lymphocytes are not infected with virus (Geisbert et al., 2000). In patients in which this has been investigated encephalitis was detected (Shifflett and Marzi, 2019). Based on in vitro studies with Egyptian fruit bat and human cell lines, the innate immune response upon Marburg virus infection is much stronger in Egyptian fruit bats than in humans (Kuzmin et al., 2017). This was suggested to be a reason for the difference in pathologic outcome of the infection.

**6. Lassa virus (ambisense, single-stranded RNA virus, genus Mammarenavirus, order Bunyavirales) infection in Natal multimammate rats (*Mastomys natalensis*; order Rodentia), versus Lassa virus infection in humans.**

**Attachment site:** In general, Lassa virus and other arenaviruses attach to dystroglycan. This is a cellular receptor for extracellular matrix protein that is conserved in mammals. Dystroglycan is found in most tissues. The Lassa virus surface protein that binds the host receptor (the glycoprotein) mimics host-derived extracellular matrix proteins, which are tissue and likely host-specific. Attachment frequently does not lead to virus entry. Virus entry, rather than virus attachment, mainly influences host and cell type tropism, but its importance in host, tissue and cell tropism has not been well studied. In addition, Lassa virus has been found to attach to multiple other receptors (Torriani et al., 2017), but its relevance for the pathogenesis in different species is not known.

**Entry:** In *Mastomys spp.*, like the Natal multimammate rat, Lassa virus is suggested to be transmitted vertically via the placenta (Asogun et al., 2019), and by contact with urine or faeces of conspecifics. It is not known what the general tissue site, or cell type of the start of infection is. Humans mainly acquire Lassa virus infection through indirect or direct contact with urine, faeces, blood or meat of different species of mastomys rats. Besides direct contact, inhalation of aerosols containing viral particles originating from rat urine or faeces has also been suggested as a route of infection. In humans, dendritic cells and macrophages are speculated to be important cells for the virus to enter a new hosed based on in vitro studies and in vivo studies in macaques (Baize et al., 2004, Hensley et al., 2011). It is not clear how, or in which tissues, these cells become infected.

**Dissemination:** In both infected Natal multimammate rats and humans, viraemia occurs, and this seems to be the main route for viral dissemination. Virus can be titrated from serum in humans, suggesting viraemia can occur cell-free. It is not clear what the main cell type is that is responsible for the viraemia (Yun and Walker, 2012).

**Amplification:** In Natal multimammate rats an important site for viral replication is the brain, and in addition lymph nodes, liver, spleen, lung and kidney (Walker et al., 1975, Karan et al., 2019). In experimentally infected Natal multimammate rats viral antigen was shown in undefined cells in the brain, lymph nodes, thymus, and kidneys; in hepatocytes in the liver and in epithelial cells of the urinary bladder (Walker et al., 1975). In humans infected with Lassa virus important sites for viral replication are lymph nodes, liver, kidney and spleen. In species used to model human Lassa fever infectious virus can be cultured from virtually all tissues though the cell types in which viral replication occurs in these organs are not identified. In liver, mainly hepatocytes, and potentially some Kupffer cells have been determined to be infected with virus (Jahrling et al., 1980).

**Exit:** Infected Natal multimammate rats shed virus via urine, faeces and saliva for months (74 days in an experimental study), if not lifelong. Undefined virus-infected cells have been detected in the kidneys of the rats, as well as epithelial cells in the bladder (Walker et al., 1975), therefore these sites could be speculated to be involved in excretion of virus via the urine. The tissues or cell types that are involved in excretion of virus via other routes are not known. Infected humans shed virus via urine and semen, and in the case of abortion (Lassa virus infection is a common cause for abortion in endemic areas), in placental fluids. In aborted fetuses, virus is present in the blood. In humans that recover from the disease, urine and semen can contain virus weeks after the recovery. There has been one case of sexual transmission months after recovery of the disease (Asogun et al., 2019). It is not clear which tissues and cell types are mainly involved in the production of the excreted virus. In experimental infections in macaques, used to model the human disease, kidneys had high viral titers, while testes were not investigated for viral presence (Jahrling et al., 1980, Callis et al., 1982). Urinary excretion in monkey models for the human disease does not seem to occur (Callis et al., 1982). Thus, it is not known which cell types are mainly involved in excretion of virus in urine and semen.

**Outcome:** In experimentally infected, neonatal and adult Natal multimammate rats, Lassa virus causes a chronic, if not persistent infection, without any apparent disease (Walker et al., 1975). Virus antigen has been detected in bladder epithelium, hepatocytes and megakaryocytes. In thymus, lymph nodes, kidney and brain viral antigen has also been detected but the cell type was not identified. Brain has been examined for the presence of a tissue response, and some animals with viral antigen in the brain develop a mild or moderate meningoencephalitis, not further characterized (Walker et al., 1975, Karan et al., 2019). The severity of disease in humans infected with Lassa virus ranges from asymptomatic infection to a severe acute, sometimes haemorrhagic systemic disease, with a case fatality rate of 15-20%. A wide range of clinical signs has been described (McLay et al., 2014). Some infected people that recover from disease, in which virus is absent within the blood, will still have virus in their central nervous tissue, and will shed virus via urine and semen (Asogun et al., 2019) suggesting chronic infections can occur in humans. The main lesion detected in fatal human cases is hepatocellular necrosis. In addition, there is splenic necrosis, adrenocortical necrosis, mild mononuclear interstitial myocarditis, and alveolar edema (Yun and Walker, 2012). Brain lesions have not been characterized. The association between the lesions and viral antigen presence does not seem to be well described in humans. In monkeys used to model human Lassa virus infection, necrosis was associated with antigen-positive cells. In liver, hepatocytes, and likely Kupffer cells contained viral antigen. In lungs, alveolar macrophages and cells of unknown type in the interstitium contained viral antigen. In adrenals, epithelial cells of the zona glomerulosa and zona fasciculata contained viral antigen. In lymph nodes and in red pulp of the spleen, macrophages contained viral antigen (Jahrling et al., 1980). In the brains, macrophages and endothelial cells contained antigen, but neurons did not (Hensley et al., 2011). In humans that die of infection, microscopic lesions are usually not severe enough to explain the cause of death. The virus infection does not cause a cytokine storm, or activation of macrophages as is seen in other haemorrhagic fevers like Ebola. It is currently suggested death is due to increased permeability of blood vessel walls, due to infection of endothelial cells and thrombocytopenia, with the absence of lesions in blood vessels (McLay et al., 2014).

**7. Highly pathogenic avian influenza (HPAI) virus H5N1 (negative sense, single-stranded RNA virus, genus Alphainfluenzavirus, order Articulavirales) infection in chickens versus highly pathogenic avian influenza virus H5N1 infection in humans**

**Attachment site:** HPAI H5N1 virus attaches to alpha-2,3-linked sialic acids on cells of both chickens and humans. Alpha 2,3 sialic acids are present on ciliated epithelia. They attract water and keep epithelial linings moist. There are species differences in the tissue expression of alpha-2,3-linked sialic acids. In chickens they are expressed on ciliated epithelial in the upper respiratory tract. In humans the alpha-2,3-linked sialic acids are present predominantly on non-ciliated epithelial cells in the deeper airways. This species difference between chickens and humans is suggested to be, partly, the explanation for the difference in pathogenesis of the disease in chickens and humans (Kumlin et al., 2008).

**Entry:** In chickens, HPAI H5N1 virus is mainly transmitted via the air. The infection in chickens and other Galliformes starts in the nasal epithelial cells, tracheal epithelial cells and epithelial cells of air capillaries (the bird equivalent of the human type I and II pneumocytes) (Hagag et al., 2015). Humans acquire HPAI H5N1 virus infection mainly via contact with an infected chicken. The infection generally starts in the respiratory tract. HPAI H5N1 virus infects ciliated tracheal epithelial cells, non-ciliated tracheal epithelial, and type II pneumocytes in humans. In both species the virus shows tropism for epithelial cells of the respiratory tract and uses these cells to invade the host. There is also a difference. In galliformes, but not in humans, nasal epithelial cells or upper respiratory tract infection occurs. This difference is thought to be due to the lack of alpha 2,3 sialic acids in human upper respiratory tract. These alpha 2,3 sialic acids are present in the upper respiratory tract of galliformes. This difference, this lack of attachment of the upper respiratory tract in humans, has been speculated to be a reason for the inefficient transmission of HPAI H5N1 virus from human to human (Kuiken et al., 2010).

**Dissemination:** In chickens and other Galliformes, HPAI H5N1 virus disseminates from the respiratory epithelial cells to the vascular and lymphatic systems, likely due to damage of the epithelial and endothelial barrier and infection of endothelial cells. In the blood the virus can replicate in macrophages and heterophils (the bird equivalent of the neutrophil). Thus, there is both cell free and intracellular viraemia (Hagag et al., 2015). In humans, HPAI H5N1 virus usually remains within the respiratory tract. However, it can disseminate from the respiratory epithelial cells in the lungs to extra-respiratory tissues, most likely by viraemia (Peiris et al., 2007). The occurrence of viraemia is based on the pattern of infection in extra-respiratory tissues. Observing infectious virus in blood or serum is rare (Chutinimitkul et al., 2006), and it is not known whether the virus infects cells in the blood.

**Amplification:** In chickens there is not one tissue or cell type specifically responsible for virus amplification as the virus infects so many different cell types. In humans, HPAI H5N1 virus replication occurs mainly in the lungs, in bronchiolar epithelium and type II pneumocytes (van Riel et al., 2009).

**Exit:** In chickens, transmission may occur via different routes. It may occur via direct or indirect contact with infected poultry. Indirect contact includes via contaminated equipment, clothing, shoes, flies, or rodents. It also may occur via contaminated dust in the air, or via contaminated water. Infected chickens excrete virus from the respiratory tract, digestive tract and skin. The most likely sources of excreted virus are epithelial cells in the respiratory tract, intestinal epithelial cells in the digestive tract, and feather follicle epithelial cells in the skin. From these excretion sites the virus relatively easily spreads via fomites (clothing, flies, rodents) (Spekreijse et al., 2011). In humans, virus is mainly excreted from the respiratory tract and digestive tract (Writing Committee of the Second World Health Organization Consultation on Clinical Aspects of Human Infection with Avian Influenza et al., 2008). However, all bodily fluids should be considered potentially infectious (Peiris et al., 2007). In the respiratory tract, the respiratory epithelial cells of the deeper airways are the most likely source of virus.

**Outcome:** Chickens naturally and experimentally infected with HPAI H5N1 virus develop a severe systemic disease, with mortality rates of 10 to 70% depending on the setting (Akanbi and Taiwo, 2014). Virus antigen associated with lesions can be found in almost all tissues. Lesions are characterized mainly by necrosis, oedema and haemorrhage (van Riel et al., 2009, Hagag et al., 2015). Humans infected with HPAI H5N1 virus develop a severe acute respiratory disease, with a case fatality rate of 60% . Virus antigen can be detected in in bronchiolar epithelium and type II pneumocytes. In general influenza A virus infection of the deeper airways at an early stage causes a pneumonia, characterized by necrosis of bronchiolar and alveolar epithelium, alveolar oedema, haemorrhage and hyaline membranes, with infiltration of some neutrophils and fewer eosinophils. Later pneumonia is characterized by re-epithelization (type II pneumocytes hyperplasia), interstitial fibrosis of alveolar septa and infiltration with lymphocytes and plasma cells (Kuiken and Taubenberger, 2008).

**8. Monkeypox virus (double stranded DNA, genus Orthopoxvirus, order Chitovirales) infection in African rope squirrels (*Funisciurus spp.*; order Rodentia), versus monkeypox virus infection in humans**

Thus far, monkeypox virus has been detected in diverse animal species: squirrels (rope and tree), rats, striped mice, dormice, and monkeys. Rodents are thought to be the main natural reservoir for the virus (Petersen et al., 2019). Of these, mainly for rope squirrels additional data regarding the virus infection is available.

**Attachment site:** Attachment of monkeypox virus is strain- and cell-type-dependent. Glycosaminoglycans might be involved (based on vaccinia virus). The exact attachment receptors on rope squirrel hosts, and human host cells are not known. In general, poxvirus cell-tropism depends on intracellular events downstream of virus binding and entry, rather than on specific host receptors (McFadden, 2005), but its importance in host, tissue and cell tropism has not been well studied.

**Entry:** For rope squirrels, the usual route of natural transmission is not known, however, experimental intranasal and intradermal inoculation led to monkeypox virus infection. The infection started in the epithelial cells at the site of inoculation (in nose and skin) (Falendysz et al., 2017). For humans there are supposedly two main routes of transmission: via direct contact and via the upper respiratory tract. If infection starts via direct contact, keratinocytes of the stratum basale, of the stratum spinosum, and dermal fibroblasts and dendritic cells are the first cells to become infected. If infection starts via the upper respiratory tract mucosal epithelial cells are the first cell to become infected.

**Dissemination:** In rope squirrels monkeypox virus most likely disseminates via regional lymphatics into the bloodstream, this is based on the detection of monkeypox induced lesions away from the site of inoculation (Falendysz et al., 2017). In humans, based on studies in macaques, monkeypox virus disseminates via regional lymphatics to the bloodstream (Cann et al., 2013). There is a knowledge gap for both rope squirrels and humans how virus gets from the original site of infection (epidermal or mucosal epithelial cells and dendritic cells) to the lymph and blood, and whether virus in lymph and blood is within (monocytic) cells or if virus occurs free in the blood.

**Amplification:** In rope squirrels infected with monkeypox virus, the lungs, presumably epithelial cells in the lungs, are the site where amplification of virus occurs mainly (Falendysz et al., 2017). The lungs could be infected via the air after start of the infection in the upper respiratory tract, or via the blood (cell-bound or free) when infection started in the skin. In model species for human monkeypox virus infection (macaques), multiple organs amplify virus, including lung, liver, spleen, and lymph nodes (Cann et al., 2013). Cell type tropism in lung is likely epithelial, in liver, spleen and lymph nodes it is likely monocytic (Cann et al., 2013, Saijo et al., 2009).

**Exit:** Rope squirrels excrete virus via oral, nasal, rectal, ocular, and skin lesion secretions. Epithelial cells in the epidermis and tongue mucosa are infected with virus, explaining oral and skin excretion. The source of virus for excretion of virus via the other routes is not clear (Falendysz et al., 2017). In humans infected with monkeypox virus, viral excretion can occur from skin vesicles. In these lesions, keratinocytes of the stratum basale and the stratum spinosum are infected and the likely source for the viral excretion (Cann et al., 2013).

**Outcome:** Rope squirrels experimentally infected had a high case fatality rate (total n=8; CFR 50-75%). The skin and oral mucosa showed, associated with the presence of virus, epithelial hyperplasia and necrosis, with lymphoplasmacytic dermatitis and stomatitis. Lesions in other organs were not associated with viral antigen. Kidneys had widespread renal tubular degeneration, with lymphoplasmacytic infiltration. Hearts had multifocal chronic lymphoplasmacytic pericarditis, myocarditis and endocarditis. It is unknown whether this experimental infection mimics natural productive infection in rope squirrels. The seroprelalence of 25% in some populations (Falendysz et al., 2017) could suggest that the disease in natural infections is less lethal than seen during the experiment. Humans infected with monkeypox virus have a severe acute systemic disease with cutaneous rash. The human disease has a case fatality rate of 10%, but the pathogenesis of this severe disease is not well described. Lesions described in model species for human monkeypox virus infection (macaques) are epithelial hyperplasia, with viral inclusions in keratinocytes, and severe necrosis in skin and oral mucosa. Vesicular lesions show spongiosis with reticular and ballooning degeneration of the epithelium and presence of multinucleated epithelial giant cells with infiltration with numerous eosinophils and neutrophils. Within the underlying dermis or submucosa there is vasculitis, and lymphoplasmacytic infiltration. In other organs (lungs, lymph nodes, spleen, and liver) the lesions are also mainly necrotizing, and they are associated with the presence of the virus (Cann et al., 2013).

**9. Nipah virus (negative sense, single-stranded RNA virus, genus Henipavirus, order Mononegavirales) infection in flying foxes (*Pteropus spp*.; order Chiroptera), versus Nipah virus infection in humans**

**Attachment site:** Nipah virus attaches to ephrin B2 and ephrin B3. Ephrins are ligands for tyrosine kinase receptors that are generally present on cell membranes. Their function is broad and mainly related to cell-cell interactions. In Pteropidae, the distribution of ephrins has not been investigated, but ephrins are well conserved in mammals including in Pteropidae (Bossart et al., 2009, Laing et al., 2019). In humans, ephrin B2 is present on endothelial cells, type II pneumocytes, epithelial cells and in small amounts on neurons, and ephrin B3 is present also on neurons (Xu and Henkemeyer, 2012). It is not known whether there are species differences in ephrin B2 and ephrin B3 distribution, and whether this might be one of the underlying causes for differences in the pathogenesis between Nipah virus infection in Pteropidae and humans.

**Entry:** In Pteropidae, Nipah virus is likely mainly transmitted via direct contact, or indirect contact with excreta of infected bats. Subcutaneous inoculation resulted in a productive infection in an experiment performed with grey-headed flying foxes (*Pteropus poliocephalus*), suggesting virus might be transmitted via biting (Middleton et al., 2007b). It is not known in which cell types the Nipah virus infection starts. Humans acquire Nipah virus infection via direct contact with infected intermediate hosts (pigs), infected humans, or indirect contact with urine of infected Pteropidae, likely via inhalation of aerosols or respiratory particles containing viral particles. The first cells that become infected are thought to be macrophages, bronchiolar epithelial cells, and type II pneumocytes (Escaffre et al., 2013).

**Dissemination:** In Pteropidae, virus dissemination is likely via blood, though direct evidence for this is still lacking. In humans a viraemia occurs, and virus disseminates from the initial site of replication to multiple tissues via the blood (Wong et al., 2002a). For both Pteropidae and humans it is unknown whether virus in blood is within (monocytic) cells or if virus occurs in the blood cell free.

**Amplification:** In Pteropidae, virus likely replicates in endothelial cells of arteries and veins, though direct evidence for this is still lacking (de Wit and Munster, 2015). In humans, virus replication occurs in endothelial cells of small arteries, arterioles, capillaries, and venules mostly in the brain, and less in lungs, kidneys and heart (Wong et al., 2002a).

**Exit:** In naturally infected bats (*Pteropus vampyrus* and *Pteropus hypomelanus*), virus was isolated from, or detected in, oro-pharyngeal swabs, partly eaten fruits (suggesting oral excretion) and urine (Chua et al., 2002, Sendow et al., 2013). In experimentally infected *Pteropus poliocephalus*, virus was detected in urine, but not in oro-pharyngeal swabs, conjunctival, rectal, and nasal swabs, nor in blood (Middleton et al., 2007a). In experimentally infected *Pteropus vampyrus*, virus was detected in a rectal and an oro-pharyngeal swab (Halpin et al., 2011). It has been speculated that, similar to Hendra virus in Pteropid bats, Nipah virus can be transmitted during parturition, thus via excretion via the female genital tract, but evidence for this is still lacking (Dimitrov and Wang, 2007). In humans, Nipah virus can be excreted from the throat, nose and via urine (Chua et al., 2002). The origin of this virus could be virus in the blood, via serum leakage in named sites.

**Outcome:** Pteropidae have not been described to develop symptoms when infected with Nipah virus. As Nipah infected cells could not be detected by immunohistochemistry in a wide range of organs investigated in experimentally infected bats, it is difficult to associate lesions with infection (de Wit and Munster, 2015). People develop an acute severe respiratory, but mainly neurologic disease, with a case fatality rate of 40-70%. Lesions in lungs and brain are necrotizing vasculitis, with thrombosis, syncytia formation, and viral inclusions. Besides endothelial cells and lung epithelial cells, neurons can also become infected. In 7.5% of human cases, there is late onset encephalitis, weeks to years after the initial symptoms (Wong et al., 2002b, de Wit and Munster, 2015).

**10. Middle East respiratory syndrome (MERS) virus (positive sense, single stranded RNA virus, genus Betacoronavirus, order Nidovirales) infection in dromedaries (*Camelus dromedarius*; order Artiodactyla) versus MERS virus infection in humans**

**Attachment site:** MERS virus attaches to dipeptidyl peptidase 4 (DPP4) and alpha-2,3-linked sialic acids. DPP4 as a transmembrane protein is present on the surface of most cell types, and is associated with immune regulation, signal transduction, and apoptosis. Alpha-2,3-linked sialic acids are present on ciliated epithelia, where they attract water and keep epithelial linings moist. There are species differences in the expression of DPP4 and alpha-2,3-linked sialic acids. In dromedaries, they are present on upper respiratory tract epithelial cells of nasal turbinates and the larynx. In humans, they are present on type II pneumocytes. This species difference between dromedaries and humans is suggested to partly explain the difference in pathogenesis of the disease in dromedaries and humans (Widagdo et al., 2016).

**Entry:** In dromedaries, MERS virus is suggested to be mainly transmitted via close contact or fomites. The infection in dromedaries starts in the epithelium of the nasal turbinates and larynx (Haverkamp et al., 2018). In humans, MERS virus infection is mainly transmitted by close contact with infected camels or humans. The infection in humans likely starts in the type II pneumocytes (de Wit et al., 2016).

**Dissemination:** Both in dromedaries and humans, the infection is usually limited to the initial site of infection; no further dissemination to other organ systems occurs (Haverkamp et al., 2018).

**Amplification:** The amplification cell type is the same as the entry cell type for both dromedaries and humans.

**Exit:** In dromedaries, transmission likely mainly occurs via direct contact and fomites. Naturally and experimentally infected dromedaries excrete virus via nose exudate, and less frequently faeces (Raj et al., 2014, Hemida et al., 2014, Haagmans et al., 2016). Nasal epithelial cells are the most likely source of the excreted virus in the nose (Haverkamp et al., 2018). The origin of the virus detected in faeces is not clear (Hemida et al., 2014, Haverkamp et al., 2018). In humans, virus is mainly transmitted during severe clinical disease, when patients are already in the hospital (de Wit et al., 2016). Infected humans excrete virus via sputum (Zaki et al., 2012). Type II pneumocytes in the deeper airways are the most likely source of this virus.

**Outcome:** Dromedaries naturally and experimentally infected with MERS virus develop mild disease with exudation from the nose (Haverkamp et al., 2018, Alharbi et al., 2020). Virus has been detected by immunohistochemistry in epithelial cells in nasal turbinates and trachea, as well as in macrophages in markedly infected tissues. Lesions associated with virus antigen detection are acute mild rhinitis, tracheitis, and bronchitis, characterized by erosion of epithelium and infiltration with neutrophils, and increased mucus presence on surfaces (Haverkamp et al., 2018). Humans develop a severe respiratory disease with a case fatality rate of 35%. Virus has been detected in lungs in pneumocytes, syncytial cells and macrophages, in macrophages away from the lungs (in skeletal muscle of the leg of a patient), and in one patient in kidney in proximal tubular epithelial cells. Lesions associated with virus antigen detection are diffuse alveolar damage in the lungs, lymphocytic myositis in skeletal muscle, while in kidneys epithelial cells showed mild degeneration (Alsaad et al., 2018, Ng et al., 2016). In macaques used to model the human disease, viral antigen has been detected in type I and type II pneumocytes, and macrophages. Lesions are pneumonia characterized by thickened alveolar septa with oedema, fibrin and few macrophages and neutrophils, intraluminal alveolar macrophages, neutrophils and multinucleated giant cells, fibrin and sloughed epithelial cells. Later there is type II pneumocyte hyperplasia with alveolar oedema, fibrin deposition and hyaline membranes (van den Brand et al., 2015, de Wit et al., 2016). Human T cells can be infected with MERS virus, but the clinical relevance of this is not clear (Haverkamp et al., 2018).

**11. Simian immunodeficiency virus (SIV; positive sense, single stranded RNA virus, genus Lentivirus, order Ortervirales) infection in common chimpanzees (*Pan troglodytes*; order Primates) versus human immunodeficiency virus-1 (HIV-1) infection in humans**

**Attachment site:** Both SIV and HIV-1 attach to CD4 and the chemokine coreceptors CCR5 or CXCR4 in their hosts. The CD4 receptor is different in humans and chimpanzees, and the differences are suggested to affect the ability of the viruses to infect the host (Bibollet-Ruche et al., 2019).

**Entry:** In chimpanzees, the routes of transmission of SIV infection between chimpanzees are not known, and there is little evidence that mucosal transmission is the dominant route for lentiviruses in primate populations in nature (Weber, 2001, Heeney et al., 2006). In humans, HIV-1 infection is mainly transmitted via mucosa mainly during sexual contacts. Another route is via injection when needles are shared between drug users. For both chimpanzees and humans, it is suggested that infection starts with free virus attaching to DC-SiGN receptors on Langerhans cells, which are a specialized mucosal macrophage. The Langerhans cells subsequently attract CD4+ T cells, to which they present the DC-SiGN receptors with the viruses attached to them. The virus can then infect these CD4+ T cells, which then migrate to regional lymph nodes.

Interestingly, chimpanzee peripheral blood mononuclear cells (which also include macrophages) are considerably less susceptible to SIV (also HIV-1) infection, than human peripheral blood mononuclear cells are susceptible to HIV-1 (and also SIV) infection. The reason for this species difference in susceptibility for both lentiviruses is not clear (Sharp et al., 2005).

**Dissemination & Amplification:** Both in chimpanzees infected with SIV and in humans infected with HIV-1, virus disseminates via the blood to many distant immune sites throughout the body. Experimental infection of chimpanzees (n=6) showed SIV causes a high plasma viraemia. The exact origin of the virus is not known. Neutralizing antibodies develop after approximately four weeks (Heeney et al., 2006). For HIV-1 infection in humans this phase in the pathogenesis is called ‘plasma viraemia’, and like in SIV infection, it is associated with a large amount of virus in plasma. At the start of the viraemia, HIV-1 and SIV are both produced by the CD4+ T cells in the regional lymph nodes. Plasma viraemia occurs approximately 2 to 6 weeks after infection in HIV-1 patients, it resolves after 2-4 weeks associated with the detection of antibodies and cell mediated immune response against the virus (Weber, 2001). For both SIV infection in chimpanzees and HIV-1 infection in humans, virus will continue to be present in the blood after the initial high plasma viraemia.

In chimpanzees infected with SIV and in humans infected with HIV-1, CD4+T cells are the main cells involved in amplifying virus. For chimpanzees infected with SIV it is not known if there is a specific subtype of CD4+T cells involved (Sharp et al., 2005). In humans, memory CD4+T cells are the cell type that is most affected by the infection (Greenwood et al., 2015).

**Exit:** Chimpanzees and humans excrete virus in fluids covering mucous membranes. The virus likely originates from the blood.

**Outcome:** In both chimpanzees infected with SIV and in humans infected with HIV-1 the infection becomes persistent. In the majority of infected chimpanzees, the number of CD4+ T cells decrease initially, but restore to normal levels (Greenwood et al., 2015). While SIV replicates to titers of 10^4 to 10^5, this often is not associated with CD4+ cell decline or loss of CD4 cell function, nor clinical disease (Heeney et al., 2006). The immune activation, that is part of the pathogenesis of HIV-1 in humans, is not seen in chimpanzees infected with SIV (Heeney et al., 2006). In a minority of infected chimpanzees, CD4+ T cells do not restore to normal levels, and some develop a disease similar to acquired immune deficiency syndrome (AIDS). Lymphodepletion can be seen at autopsy. SIV infected chimpanzees have a decreased lifespan compared to non-infected chimpanzees (Sharp et al., 2005, Terio et al., 2011, Greenwood et al., 2015). In humans, the described plasma viraemia is associated with a marked loss in CD4+ T cells, but it is not known whether this is a direct or indirect effect of the virus. Usually, this initial loss in CD4+ T cells is not associated with any clinical disease. If it is, there might fever, rash, swollen and painful joints and lymph nodes, and this is suggested to be an effect of the antibodies. In only a few patients the loss of CD4+ T cells is so severe that it leads to severe transient immunosuppression with variable diseases due to opportunistic infections. After the plasma viraemic phase has resolved, CD4+ T cells partly restore their levels in the blood, to lower levels than pre-infection. HIV-1 plasma viraemia remains detectable afterwards, but the amount of virus differs a lot between individuals; the reasons for this variability are not well known. The lower the viraemia, the slower the decline of CD4+ T cells in the circulation, and the longer the period without clinical disease due to immunosuppression. It is thought that the CD4+ T cell decline in the circulation is due to decreased survival of infected CD4+ T cells. The loss of CD4+ T cells from the gut during infection has two effects upon the immune system: it depletes a major portion of the immune reserve in the form of memory CD4 T cells, and it allows microbial translocation, which establishes a state of chronic immune activation. The immune activation has many effects, e.g. it causes CD4+ T cell proliferation so that levels are partly restored.

The majority of infected humans develop AIDS, due to loss of CD4+ T cells. A minority of infected humans do not develop disease (Weber, 2001). In summary, initially SIV and HIV-1 infections are similar. Later, they become different in that the HIV-1 infection in humans causes immune activation, progressive CD4+ T-cell loss, and the development of acquired immunodeficiency syndrome (AIDS).

**References**

AKANBI, O. B. & TAIWO, V. O. 2014. Mortality and pathology associated with highly pathogenic avian influenza H5N1 outbreaks in commercial poultry production systems in Nigeria. *Int Sch Res Notices,* 2014**,** 415418.

ALHARBI, N. K., IBRAHIM, O. H., ALHAFUFI, A., KASEM, S., ALDOWERIJ, A., ALBRAHIM, R., ABU-OBAIDAH, A., ALKARAR, A., BAYOUMI, F. A., ALMANSOUR, A. M., ALDUBAIB, M., AL-ABDELY, H. M., BALKHY, H. H. & QASIM, I. 2020. Challenge infection model for MERS-CoV based on naturally infected camels. *Virol J,* 17**,** 77.

ALSAAD, K. O., HAJEER, A. H., AL BALWI, M., AL MOAIQEL, M., AL OUDAH, N., AL AJLAN, A., ALJOHANI, S., ALSOLAMY, S., GMATI, G. E., BALKHY, H., AL-JAHDALI, H. H., BAHAROON, S. A. & ARABI, Y. M. 2018. Histopathology of Middle East respiratory syndrome coronovirus (MERS-CoV) infection - clinicopathological and ultrastructural study. *Histopathology,* 72**,** 516-524.

ASOGUN, D. A., GUNTHER, S., AKPEDE, G. O., IHEKWEAZU, C. & ZUMLA, A. 2019. Lassa Fever: Epidemiology, clinical features, diagnosis, management and prevention. *Infect Dis Clin North Am,* 33**,** 933-951.

BAILEY, C. C. & MILLER, A. D. 2012. Ulcerative cheilitis in a rhesus macaque. *Vet Pathol,* 49**,** 412-5.

BAIZE, S., KAPLON, J., FAURE, C., PANNETIER, D., GEORGES-COURBOT, M. C. & DEUBEL, V. 2004. Lassa virus infection of human dendritic cells and macrophages is productive but fails to activate cells. *J Immunol,* 172**,** 2861-9.

BIBOLLET-RUCHE, F., RUSSELL, R. M., LIU, W., STEWART-JONES, G. B. E., SHERRILL-MIX, S., LI, Y., LEARN, G. H., SMITH, A. G., GONDIM, M. V. P., PLENDERLEITH, L. J., DECKER, J. M., EASLICK, J. L., WETZEL, K. S., COLLMAN, R. G., DING, S., FINZI, A., AYOUBA, A., PEETERS, M., LEENDERTZ, F. H., VAN SCHIJNDEL, J., GOEDMAKERS, A., TON, E., BOESCH, C., KUEHL, H., ARANDJELOVIC, M., DIEGUEZ, P., MURAI, M., COLIN, C., KOOPS, K., SPEEDE, S., GONDER, M. K., MULLER, M. N., SANZ, C. M., MORGAN, D. B., ATENCIA, R., COX, D., PIEL, A. K., STEWART, F. A., NDJANGO, J. N., MJUNGU, D., LONSDORF, E. V., PUSEY, A. E., KWONG, P. D., SHARP, P. M., SHAW, G. M. & HAHN, B. H. 2019. CD4 receptor diversity in chimpanzees protects against SIV infection. *Proc Natl Acad Sci U S A,* 116**,** 3229-3238.

BOSSART, K. N., ZHU, Z., MIDDLETON, D., KLIPPEL, J., CRAMERI, G., BINGHAM, J., MCEACHERN, J. A., GREEN, D., HANCOCK, T. J., CHAN, Y. P., HICKEY, A. C., DIMITROV, D. S., WANG, L. F. & BRODER, C. C. 2009. A neutralizing human monoclonal antibody protects against lethal disease in a new ferret model of acute Nipah virus infection. *PLoS Pathog,* 5.

BRUMMER-KORVENKONTIO, M., VAHERI, A., HOVI, T., VON BONSDORFF, C. H., VUORIMIES, J., MANNI, T., PENTTINEN, K., OKER-BLOM, N. & LAHDEVIRTA, J. 1980. Nephropathia epidemica: detection of antigen in bank voles and serologic diagnosis of human infection. *J Infect Dis,* 141**,** 131-4.

CALLIS, R. T., JAHRLING, P. B. & DEPAOLI, A. 1982. Pathology of Lassa virus infection in the rhesus monkey. *Am J Trop Med Hyg,* 31**,** 1038-45.

CANN, J. A., JAHRLING, P. B., HENSLEY, L. E. & WAHL-JENSEN, V. 2013. Comparative pathology of smallpox and monkeypox in man and macaques. *J Comp Pathol,* 148**,** 6-21.

CARLSON, C. S., O'SULLIVAN, M. G., JAYO, M. J., ANDERSON, D. K., HARBER, E. S., JEROME, W. G., BULLOCK, B. C. & HEBERLING, R. L. 1997. Fatal disseminated cercopithecine herpesvirus 1 (herpes B infection in cynomolgus monkeys (*Macaca fascicularis*). *Vet Pathol,* 34**,** 405-14.

CHARLTON, K. M. & CASEY, G. A. 1981. Experimental rabies in skunks: persistence of virus in denervated muscle at the inoculation site. *Can J Comp Med,* 45**,** 357-62.

CHOI, Y., KWON, Y. C., KIM, S. I., PARK, J. M., LEE, K. H. & AHN, B. Y. 2008. A hantavirus causing hemorrhagic fever with renal syndrome requires gC1qR/p32 for efficient cell binding and infection. *Virology,* 381**,** 178-83.

CHUA, K. B., LEK KOH, C., HOOI, P. S., WEE, K. F., KHONG, J. H., CHUA, B. H., CHAN, Y. P., LIM, M. E. & LAM, S. K. 2002. Isolation of Nipah virus from Malaysian Island flying-foxes. *Microbes Infect,* 4**,** 145-151.

CHUTINIMITKUL, S., BHATTARAKOSOL, P., SRISURATANON, S., EIAMUDOMKAN, A., KONGSOMBOON, K., DAMRONGWATANAPOKIN, S., CHAISINGH, A., SUWANNAKARN, K., CHIEOCHANSIN, T., THEAMBOONLERS, A. & POOVORAWAN, Y. 2006. H5N1 influenza A virus and infected human plasma. *Emerg Infect Dis,* 12**,** 1041-3.

DE WIT, E. & MUNSTER, V. J. 2015. Animal models of disease shed light on Nipah virus pathogenesis and transmission. *J Pathol,* 235**,** 196-205.

DE WIT, E., VAN DOREMALEN, N., FALZARANO, D. & MUNSTER, V. J. 2016. SARS and MERS: recent insights into emerging coronaviruses. *Nat Rev Microbiol,* 14**,** 523-34.

DIMITROV, D. S. & WANG, L. F. 2007. In utero transmission of Nipah virus: role played by pregnancy and vertical transmission in Henipavirus epidemiology. *J Infect Dis,* 196**,** 807-9.

EBERLE, R. & JONES-ENGEL, L. 2018. Questioning the extreme neurovirulence of monkey B virus (Macacine alphaherpesvirus 1). *Adv Virol,* 2018**,** 5248420.

ERMONVAL, M., BAYCHELIER, F. & TORDO, N. 2016. What do we know about how hantaviruses interact with their different hosts? *Viruses,* 8, 223.

ESCAFFRE, O., BORISEVICH, V. & ROCKX, B. 2013. Pathogenesis of Hendra and Nipah virus infection in humans. *J Infect Dev Ctries,* 7**,** 308-11.

EVANDER, M., ERIKSSON, I., PETTERSSON, L., JUTO, P., AHLM, C., OLSSON, G. E., BUCHT, G. & ALLARD, A. 2007. Puumala hantavirus viremia diagnosed by real-time reverse transcriptase PCR using samples from patients with hemorrhagic fever and renal syndrome. *J Clin Microbiol,* 45**,** 2491-7.

FALENDYSZ, E. A., LOPERA, J. G., DOTY, J. B., NAKAZAWA, Y., CRILL, C., LORENZSONN, F., KALEMBA, L. N., RONDEROS, M. D., MEJIA, A., MALEKANI, J. M., KAREM, K., CARROLL, D. S., OSORIO, J. E. & ROCKE, T. E. 2017. Characterization of Monkeypox virus infection in African rope squirrels (*Funisciurus sp.*). *PLoS Negl Trop Dis,* 11**,** e0005809.

GAMINO, V. & HOFLE, U. 2013. Pathology and tissue tropism of natural West Nile virus infection in birds: a review. *Vet Res,* 44**,** 39.

GAVRILOVSKAYA, I. N., PERESLENI, T., GEIMONEN, E. & MACKOW, E. R. 2002. Pathogenic hantaviruses selectively inhibit beta3 integrin directed endothelial cell migration. *Arch Virol,* 147**,** 1913-31.

GEISBERT, T. W., HENSLEY, L. E., GIBB, T. R., STEELE, K. E., JAAX, N. K. & JAHRLING, P. B. 2000. Apoptosis induced in vitro and in vivo during infection by Ebola and Marburg viruses. *Lab Invest,* 80**,** 171-86.

GODOY, P., MARSAC, D., STEFAS, E., FERRER, P., TISCHLER, N. D., PINO, K., RAMDOHR, P., VIAL, P., VALENZUELA, P. D., FERRES, M., VEAS, F. & LOPEZ-LASTRA, M. 2009. Andes virus antigens are shed in urine of patients with acute hantavirus cardiopulmonary syndrome. *J Virol,* 83**,** 5046-55.

GREENWOOD, E. J., SCHMIDT, F., KONDOVA, I., NIPHUIS, H., HODARA, V. L., CLISSOLD, L., MCLAY, K., GUERRA, B., REDROBE, S., GIAVEDONI, L. D., LANFORD, R. E., MURTHY, K. K., ROUET, F. & HEENEY, J. L. 2015. Simian immunodeficiency virus infection of chimpanzees (*Pan troglodytes*) shares features of both pathogenic and non-pathogenic lentiviral infections. *PLoS Pathog,* 11**,** e1005146.

GROEN, J., BRUIJN, J. A., GERDING, M. N., JORDANS, J. G., MOLL VAN CHARANTE, A. W. & OSTERHAUS, A. D. 1996. Hantavirus antigen detection in kidney biopsies from patients with nephropathia epidemica. *Clin Nephrol,* 46**,** 379-83.

GUPTA, R. K. & GUPTA, G. S. 2012. DC-SIGN family of receptors. *Animal Lectins: Form, Function and Clinical Applications.* Vienna: Springer Vienna.

HAAGMANS, B. L., VAN DEN BRAND, J. M., RAJ, V. S., VOLZ, A., WOHLSEIN, P., SMITS, S. L., SCHIPPER, D., BESTEBROER, T. M., OKBA, N., FUX, R., BENSAID, A., SOLANES FOZ, D., KUIKEN, T., BAUMGARTNER, W., SEGALES, J., SUTTER, G. & OSTERHAUS, A. D. 2016. An orthopoxvirus-based vaccine reduces virus excretion after MERS-CoV infection in dromedary camels. *Science,* 351**,** 77-81.

HAGAG, I. T., MANSOUR, S. M., ZHANG, Z., ALI, A. A., ISMAIEL EL, B. M., SALAMA, A. A., CARDONA, C. J., COLLINS, J. & XING, Z. 2015. Pathogenicity of highly pathogenic avian influenza virus H5N1 in naturally infected poultry in Egypt. *PLoS One,* 10**,** e0120061.

HÄGELE, S., NUSSHAG, C., MULLER, A., BAUMANN, A., ZEIER, M. & KRAUTKRAMER, E. 2021. Cells of the human respiratory tract support the replication of pathogenic Old World orthohantavirus Puumala. *Virol J,* 18**,** 169.

HALPIN, K., HYATT, A. D., FOGARTY, R., MIDDLETON, D., BINGHAM, J., EPSTEIN, J. H., RAHMAN, S. A., HUGHES, T., SMITH, C., FIELD, H. E., DASZAK, P. & HENIPAVIRUS ECOLOGY RESEARCH, G. 2011. Pteropid bats are confirmed as the reservoir hosts of henipaviruses: a comprehensive experimental study of virus transmission. *Am J Trop Med Hyg,* 85**,** 946-51.

HARDESTAM, J., KARLSSON, M., FALK, K. I., OLSSON, G., KLINGSTROM, J. & LUNDKVIST, A. 2008. Puumala hantavirus excretion kinetics in bank voles (*Myodes glareolus*). *Emerg Infect Dis,* 14**,** 1209-15.

HAVERKAMP, A. K., LEHMBECKER, A., SPITZBARTH, I., WIDAGDO, W., HAAGMANS, B. L., SEGALES, J., VERGARA-ALERT, J., BENSAID, A., VAN DEN BRAND, J. M. A., OSTERHAUS, A. & BAUMGARTNER, W. 2018. Experimental infection of dromedaries with Middle East respiratory syndrome-Coronavirus is accompanied by massive ciliary loss and depletion of the cell surface receptor dipeptidyl peptidase 4. *Sci Rep,* 8**,** 9778.

HEENEY, J. L., RUTJENS, E., VERSCHOOR, E. J., NIPHUIS, H., TEN HAAFT, P., ROUSE, S., MCCLURE, H., BALLA-JHAGJHOORSINGH, S., BOGERS, W., SALAS, M., COBB, K., KESTENS, L., DAVIS, D., VAN DER GROEN, G., COURGNAUD, V., PEETERS, M. & MURTHY, K. K. 2006. Transmission of simian immunodeficiency virus SIVcpz and the evolution of infection in the presence and absence of concurrent human immunodeficiency virus type 1 infection in chimpanzees. *J Virol,* 80**,** 7208-18.

HEMIDA, M. G., CHU, D. K., POON, L. L., PERERA, R. A., ALHAMMADI, M. A., NG, H. Y., SIU, L. Y., GUAN, Y., ALNAEEM, A. & PEIRIS, M. 2014. MERS coronavirus in dromedary camel herd, Saudi Arabia. *Emerg Infect Dis,* 20**,** 1231-4.

HENSLEY, L. E., SMITH, M. A., GEISBERT, J. B., FRITZ, E. A., DADDARIO-DICAPRIO, K. M., LARSEN, T. & GEISBERT, T. W. 2011. Pathogenesis of Lassa fever in cynomolgus macaques. *Virol J,* 8**,** 205.

JACKSON, A. C. & FU, Z. F. 2013. Pathogenesis. *In:* JACKSON, A. C. (ed.) *Rabies: scientific basis of the disease and its management.* 3rd ed. Amsterdam: Academic Press.

JAHRLING, P. B., HESSE, R. A., EDDY, G. A., JOHNSON, K. M., CALLIS, R. T. & STEPHEN, E. L. 1980. Lassa virus infection of rhesus monkeys: pathogenesis and treatment with ribavirin. *J Infect Dis,* 141**,** 580-9.

JANGRA, R. K., HERBERT, A. S., LI, R., JAE, L. T., KLEINFELTER, L. M., SLOUGH, M. M., BARKER, S. L., GUARDADO-CALVO, P., ROMAN-SOSA, G., DIETERLE, M. E., KUEHNE, A. I., MUENA, N. A., WIRCHNIANSKI, A. S., NYAKATURA, E. K., FELS, J. M., NG, M., MITTLER, E., PAN, J., BHARRHAN, S., WEC, A. Z., LAI, J. R., SIDHU, S. S., TISCHLER, N. D., REY, F. A., MOFFAT, J., BRUMMELKAMP, T. R., WANG, Z., DYE, J. M. & CHANDRAN, K. 2018. Protocadherin-1 is essential for cell entry by New World hantaviruses. *Nature,* 563**,** 559-563.

JONES, M. E. B., AMMAN, B. R., SEALY, T. K., UEBELHOER, L. S., SCHUH, A. J., FLIETSTRA, T., BIRD, B. H., COLEMAN-MCCRAY, J. D., ZAKI, S. R., NICHOL, S. T. & TOWNER, J. S. 2019. Clinical, histopathologic, and immunohistochemical characterization of experimental Marburg virus infection in a natural reservoir host, the Egyptian rousette bat (*Rousettus aegyptiacus*). *Viruses,* 11.

KARAN, L., MAKENOV, M., KORNEEV, M., SACKO, N., BOUMBALY, S., BAYANDIN, R., GLADYSHEVA, A., KOUROUMA, K., TOURE, A., KARTASHOV, M., SHIPOVALOV, A., PORSHAKOV, A., KOULIBALY, M. & BOIRO, M. 2019. Lassa virus in the host rodent (*Mastomys Natalensis*) within Urban Areas of N’zerekore, Guinea. *bioRxiv***,** 616466.

KRAUTKRAMER, E. & ZEIER, M. 2008. Hantavirus causing hemorrhagic fever with renal syndrome enters from the apical surface and requires decay-accelerating factor (DAF/CD55). *J Virol,* 82**,** 4257-64.

KUIKEN, T. & TAUBENBERGER, J. K. 2008. Pathology of human influenza revisited. *Vaccine,* 26 Suppl 4**,** D59-66.

KUIKEN, T., VAN DEN BRAND, J., VAN RIEL, D., PANTIN-JACKWOOD, M. & SWAYNE, D. E. 2010. Comparative pathology of select agent influenza a virus infections. *Vet Pathol,* 47**,** 893-914.

KUMLIN, U., OLOFSSON, S., DIMOCK, K. & ARNBERG, N. 2008. Sialic acid tissue distribution and influenza virus tropism. *Influenza Other Respir Viruses,* 2**,** 147-54.

KUZMIN, I. V., SCHWARZ, T. M., ILINYKH, P. A., JORDAN, I., KSIAZEK, T. G., SACHIDANANDAM, R., BASLER, C. F. & BUKREYEV, A. 2017. Innate immune responses of bat and human cells to filoviruses: commonalities and distinctions. *J Virol,* 91.

LAFON, M. 2005. Rabies virus receptors. *J Neurovirol,* 11**,** 82-7.

LAING, E. D., NAVARATNARAJAH, C. K., CHELIOUT DA SILVA, S., PETZING, S. R., XU, Y., STERLING, S. L., MARSH, G. A., WANG, L. F., AMAYA, M., NIKOLOV, D. B., CATTANEO, R., BRODER, C. C. & XU, K. 2019. Structural and functional analyses reveal promiscuous and species specific use of ephrin receptors by Cedar virus. *Proc Natl Acad Sci U S A,* 116**,** 20707-20715.

LATUS, J., TENNER-RACZ, K., RACZ, P., KITTERER, D., CADAR, D., OTT, G., ALSCHER, M. D., SCHMIDT-CHANASIT, J. & BRAUN, N. 2014. Detection of Puumala hantavirus antigen in human intestine during acute hantavirus infection. *PLoS One,* 9**,** e98397.

LI, Z., FENG, Z. & YE, H. 1995. Rabies viral antigen in human tongues and salivary glands. *J Trop Med Hyg,* 98**,** 330-2.

MAAS, M., VAN HETEREN, M., DE VRIES, A., KUIKEN, T., HOORNWEG, T., VELDHUIS KROEZE, E. & ROCKX, B. 2019. Seoul virus tropism and pathology in naturally infected feeder rats. *Viruses,* 11, 531.

MCFADDEN, G. 2005. Poxvirus tropism. *Nat Rev Microbiol,* 3**,** 201-13.

MCLAY, L., LIANG, Y. & LY, H. 2014. Comparative analysis of disease pathogenesis and molecular mechanisms of New World and Old World arenavirus infections. *J Gen Virol,* 95**,** 1-15.

MIDDLETON, D. J., MORRISSY, C. J., VAN DER HEIDE, B. M., RUSSELL, G. M., BRAUN, M. A., WESTBURY, H. A., HALPIN, K. & DANIELS, P. W. 2007a. Experimental Nipah virus infection in pteropid bats (*Pteropus poliocephalus*). *J Comp Pathol,* 136**,** 266-72.

MIDDLETON, D. J., MORRISSY, C. J., VAN DER HEIDE, B. M., RUSSELL, G. M., BRAUN, M. A., WESTBURY, H. A., HALPIN, K. & DANIELS, P. W. 2007b. Experimental Nipah virus infection in Pteropid bats (*Pteropus poliocephalus*). *J Comp Pathol,* 136**,** 266-272.

MULLER, A., BAUMANN, A., ESSBAUER, S., RADOSA, L., KRUGER, D. H., WITKOWSKI, P. T., ZEIER, M. & KRAUTKRAMER, E. 2019. Analysis of the integrin beta3 receptor for pathogenic orthohantaviruses in rodent host species. *Virus Res,* 267**,** 36-40.

MUSTONEN, J., MAKELA, S., OUTINEN, T., LAINE, O., JYLHAVA, J., ARSTILA, P. T., HURME, M. & VAHERI, A. 2013. The pathogenesis of nephropathia epidemica: new knowledge and unanswered questions. *Antiviral Res,* 100**,** 589-604.

MUSTONEN, J., OUTINEN, T., LAINE, O., PORSTI, I., VAHERI, A. & MAKELA, S. 2017. Kidney disease in Puumala hantavirus infection. *Infect Dis (Lond),* 49**,** 321-332.

NANDA, M., CURTIN, V. T., HILLIARD, J. K., BERNSTEIN, N. D. & DIX, R. D. 1990. Ocular histopathologic findings in a case of human herpes B virus infection. *Arch Ophthalmol,* 108**,** 713-6.

NG, D. L., AL HOSANI, F., KEATING, M. K., GERBER, S. I., JONES, T. L., METCALFE, M. G., TONG, S., TAO, Y., ALAMI, N. N., HAYNES, L. M., MUTEI, M. A., ABDEL-WARETH, L., UYEKI, T. M., SWERDLOW, D. L., BARAKAT, M. & ZAKI, S. R. 2016. Clinicopathologic, immunohistochemical, and ultrastructural findings of a fatal case of Middle East respiratory syndrome coronavirus infection in the United Arab Emirates, April 2014. *Am J Pathol,* 186**,** 652-8.

NOACK, D., GOEIJENBIER, M., REUSKEN, C., KOOPMANS, M. P. G. & ROCKX, B. H. G. 2020. Orthohantavirus pathogenesis and cell tropism. *Front Cell Infect Microbiol,* 10**,** 399.

PATRUSHEVA, I., PERELYGINA, L., TORSHIN, I., LECHER, J. & HILLIARD, J. 2016. B virus (*Macacine Herpesvirus 1*) divergence: variations in glycoprotein D from clinical and laboratory isolates diversify virus entry strategies. *J Virol,* 90**,** 9420-32.

PEIRIS, J. S., DE JONG, M. D. & GUAN, Y. 2007. Avian influenza virus (H5N1): a threat to human health. *Clin Microbiol Rev,* 20**,** 243-67.

PERELYGINA, L., PATRUSHEVA, I., VASIREDDI, M., BROCK, N. & HILLIARD, J. 2015. B Virus (Macacine herpesvirus 1) Glycoprotein D is functional but dispensable for virus entry into macaque and human skin cells. *J Virol,* 89**,** 5515-24.

PEREZ-RAMIREZ, E., LLORENTE, F. & JIMENEZ-CLAVERO, M. A. 2014. Experimental infections of wild birds with West Nile virus. *Viruses,* 6**,** 752-81.

PETERSEN, E., KANTELE, A., KOOPMANS, M., ASOGUN, D., YINKA-OGUNLEYE, A., IHEKWEAZU, C. & ZUMLA, A. 2019. Human Monkeypox: Epidemiologic and clinical characteristics, diagnosis, and prevention. *Infect Dis Clin North Am,* 33**,** 1027-1043.

PETTERSSON, L., KLINGSTROM, J., HARDESTAM, J., LUNDKVIST, A., AHLM, C. & EVANDER, M. 2008. Hantavirus RNA in saliva from patients with hemorrhagic fever with renal syndrome. *Emerg Infect Dis,* 14**,** 406-11.

PIZARRO, E., NAVARRETE, M., MENDEZ, C., ZAROR, L., MANSILLA, C., TAPIA, M., CARRASCO, C., SALAZAR, P., MURUA, R., PADULA, P., OTTH, C. & RODRIGUEZ, E. M. 2019. Immunocytochemical and ultrastructural evidence supporting that andes hantavirus (ANDV) is transmitted person-to-person through the respiratory and/or salivary pathways. *Front Microbiol,* 10**,** 2992.

PREVENTION, C. F. D. C. A. 2015. *Highly pathogenic Asian avian influenza A (H5N1) in people* [Online]. Available: <https://www.cdc.gov/flu/avianflu/h5n1-people.htm> [Accessed].

RAJ, V. S., FARAG, E. A., REUSKEN, C. B., LAMERS, M. M., PAS, S. D., VOERMANS, J., SMITS, S. L., OSTERHAUS, A. D., AL-MAWLAWI, N., AL-ROMAIHI, H. E., ALHAJRI, M. M., EL-SAYED, A. M., MOHRAN, K. A., GHOBASHY, H., ALHAJRI, F., AL-THANI, M., AL-MARRI, S. A., EL-MAGHRABY, M. M., KOOPMANS, M. P. & HAAGMANS, B. L. 2014. Isolation of MERS coronavirus from a dromedary camel, Qatar, 2014. *Emerg Infect Dis,* 20**,** 1339-42.

RITCHEY, J. W., PAYTON, M. E. & EBERLE, R. 2005. Clinicopathological characterization of monkey B virus (*Cercopithecine herpesvirus 1*) infection in mice. *J Comp Pathol,* 132**,** 202-17.

ROSSITER, J. J., AC; 2013. Pathology. *In:* JACKSON, A. C. (ed.) *Rabies: scientific basis of the disease and its management third edition.* third ed. Amsterdam: Academic Press.

SAIJO, M., AMI, Y., SUZAKI, Y., NAGATA, N., IWATA, N., HASEGAWA, H., IIZUKA, I., SHIOTA, T., SAKAI, K., OGATA, M., FUKUSHI, S., MIZUTANI, T., SATA, T., KURATA, T., KURANE, I. & MORIKAWA, S. 2009. Virulence and pathophysiology of the Congo Basin and West African strains of monkeypox virus in non-human primates. *J Gen Virol,* 90**,** 2266-71.

SALVADOR, B., SEXTON, N. R., CARRION, R., JR., NUNNELEY, J., PATTERSON, J. L., STEFFEN, I., LU, K., MUENCH, M. O., LEMBO, D. & SIMMONS, G. 2013. Filoviruses utilize glycosaminoglycans for their attachment to target cells. *J Virol,* 87**,** 3295-304.

SAMPSON, B. A., AMBROSI, C., CHARLOT, A., REIBER, K., VERESS, J. F. & ARMBRUSTMACHER, V. 2000. The pathology of human West Nile virus infection. *Hum Pathol,* 31**,** 527-31.

SAMUEL, M. A. & DIAMOND, M. S. 2006. Pathogenesis of West Nile Virus infection: a balance between virulence, innate and adaptive immunity, and viral evasion. *J Virol,* 80**,** 9349-60.

SCHNELL, M. J., MCGETTIGAN, J. P., WIRBLICH, C. & PAPANERI, A. 2010. The cell biology of rabies virus: using stealth to reach the brain. *Nat Rev Microbiol,* 8**,** 51-61.

SCHUH, A. J., AMMAN, B. R., JONES, M. E., SEALY, T. K., UEBELHOER, L. S., SPENGLER, J. R., MARTIN, B. E., COLEMAN-MCCRAY, J. A., NICHOL, S. T. & TOWNER, J. S. 2017. Modelling filovirus maintenance in nature by experimental transmission of Marburg virus between Egyptian rousette bats. *Nat Commun,* 8**,** 14446.

SEJVAR, J. J. 2016. West Nile virus infection. *Microbiol Spectr,* 4.

SENDOW, I., RATNAWATI, A., TAYLOR, T., ADJID, R. M., SAEPULLOH, M., BARR, J., WONG, F., DANIELS, P. & FIELD, H. 2013. Nipah virus in the fruit bat *Pteropus vampyrus* in Sumatera, Indonesia. *PLoS One,* 8**,** e69544.

SHARP, P. M., SHAW, G. M. & HAHN, B. H. 2005. Simian immunodeficiency virus infection of chimpanzees. *J Virol,* 79**,** 3891-902.

SHIFFLETT, K. & MARZI, A. 2019. Marburg virus pathogenesis - differences and similarities in humans and animal models. *Virol J,* 16**,** 165.

SHIWA, N., KIMITSUKI, K., MANALO, D. L., INOUE, S. & PARK, C. H. 2018. A pathological study of the tongues of rabid dogs in the Philippines. *Arch Virol,* 163**,** 1615-1621.

SIRONEN, T., KLINGSTROM, J., VAHERI, A., ANDERSSON, L. C., LUNDKVIST, A. & PLYUSNIN, A. 2008. Pathology of Puumala hantavirus infection in macaques. *PLoS One,* 3**,** e3035.

SMART, N. L. & CHARLTON, K. M. 1992. The distribution of challenge virus standard rabies virus versus skunk street rabies virus in the brains of experimentally infected rabid skunks. *Acta Neuropathol,* 84**,** 501-8.

SPEKREIJSE, D., BOUMA, A., KOCH, G. & STEGEMAN, J. A. 2011. Airborne transmission of a highly pathogenic avian influenza virus strain H5N1 between groups of chickens quantified in an experimental setting. *Vet Microbiol,* 152**,** 88-95.

TERIO, K. A., KINSEL, M. J., RAPHAEL, J., MLENGEYA, T., LIPENDE, I., KIRCHHOFF, C. A., GILAGIZA, B., WILSON, M. L., KAMENYA, S., ESTES, J. D., KEELE, B. F., RUDICELL, R. S., LIU, W., PATTON, S., COLLINS, A., HAHN, B. H., TRAVIS, D. A. & LONSDORF, E. V. 2011. Pathologic lesions in chimpanzees (Pan trogylodytes schweinfurthii) from Gombe National Park, Tanzania, 2004-2010. *J Zoo Wildl Med,* 42**,** 597-607.

TORRIANI, G., GALAN-NAVARRO, C. & KUNZ, S. 2017. Lassa virus cell entry reveals new aspects of virus-host cell interaction. *J Virol,* 91.

VAN DEN BRAND, J. M., SMITS, S. L. & HAAGMANS, B. L. 2015. Pathogenesis of Middle East respiratory syndrome coronavirus. *J Pathol,* 235**,** 175-84.

VAN RIEL, D., VAN DEN BRAND, J. M., MUNSTER, V. J., BESTEBOER, T. M., FOUCHIER, R. A., OSTERHAUS, A. D. & KUIKEN, T. 2009. Pathology and virus distribution in chickens naturally infected with highly pathogenic avian influenza A virus (H7N7) During the 2003 outbreak in The Netherlands. *Vet Pathol,* 46**,** 971-6.

VANDALEN, K. K., HALL, J. S., CLARK, L., MCLEAN, R. G. & SMERASKI, C. 2013. West Nile virus infection in American Robins: new insights on dose response. *PLoS One,* 8**,** e68537.

VOUTILAINEN, L., KALLIO, E. R., NIEMIMAA, J., VAPALAHTI, O. & HENTTONEN, H. 2016. Temporal dynamics of Puumala hantavirus infection in cyclic populations of bank voles. *Sci Rep,* 6**,** 21323.

WALKER, D. H., WULFF, H., LANGE, J. V. & MURPHY, F. A. 1975. Comparative pathology of Lassa virus infection in monkeys, guinea-pigs, and *Mastomys natalensis*. *Bull World Health Organ,* 52**,** 523-34.

WEBER, J. 2001. The pathogenesis of HIV-1 infection. *Br Med Bull,* 58**,** 61-72.

WIDAGDO, W., RAJ, V. S., SCHIPPER, D., KOLIJN, K., VAN LEENDERS, G., BOSCH, B. J., BENSAID, A., SEGALES, J., BAUMGARTNER, W., OSTERHAUS, A., KOOPMANS, M. P., VAN DEN BRAND, J. M. A. & HAAGMANS, B. L. 2016. Differential expression of the Middle East respiratory syndrome coronavirus receptor in the upper respiratory tracts of humans and dromedary camels. *J Virol,* 90**,** 4838-4842.

WONG, K. T., SHIEH, W. J., KUMAR, S. & NORAIN, K. 2002a. Nipah virus infection: pathology and pathogenesis of an emerging paramyxoviral zoonosis. *The American journal of …*.

WONG, K. T., SHIEH, W. J., KUMAR, S., NORAIN, K., ABDULLAH, W., GUARNER, J., GOLDSMITH, C. S., CHUA, K. B., LAM, S. K., TAN, C. T., GOH, K. J., CHONG, H. T., JUSOH, R., ROLLIN, P. E., KSIAZEK, T. G., ZAKI, S. R. & NIPAH VIRUS PATHOLOGY WORKING, G. 2002b. Nipah virus infection: pathology and pathogenesis of an emerging paramyxoviral zoonosis. *Am J Pathol,* 161**,** 2153-67.

WRITING COMMITTEE OF THE SECOND WORLD HEALTH ORGANIZATION CONSULTATION ON CLINICAL ASPECTS OF HUMAN INFECTION WITH AVIAN INFLUENZA, A. V., ABDEL-GHAFAR, A. N., CHOTPITAYASUNONDH, T., GAO, Z., HAYDEN, F. G., NGUYEN, D. H., DE JONG, M. D., NAGHDALIYEV, A., PEIRIS, J. S., SHINDO, N., SOEROSO, S. & UYEKI, T. M. 2008. Update on avian influenza A (H5N1) virus infection in humans. *N Engl J Med,* 358**,** 261-73.

XU, N. J. & HENKEMEYER, M. 2012. Ephrin reverse signaling in axon guidance and synaptogenesis. *Semin Cell Dev Biol,* 23**,** 58-64.

YUN, N. E. & WALKER, D. H. 2012. Pathogenesis of Lassa fever. *Viruses,* 4**,** 2031-48.

ZAKI, A. M., VAN BOHEEMEN, S., BESTEBROER, T. M., OSTERHAUS, A. D. & FOUCHIER, R. A. 2012. Isolation of a novel coronavirus from a man with pneumonia in Saudi Arabia. *N Engl J Med,* 367**,** 1814-20.
